# Comparative effects of physical education interventions on motor ability and executive function in junior high school students and their interrelationships

**DOI:** 10.3389/fpubh.2026.1779737

**Published:** 2026-04-23

**Authors:** Xinyue Zhang, Ruishi Fan, Xingrui Han, Zhouhang Wu

**Affiliations:** 1 College of Physical Education and Health, East China Normal University, Shanghai, China; 2 Key Laboratory of Adolescent Health Assessment and Exercise Intervention of Ministry of Education, East China Normal University, Shanghai, China

**Keywords:** adolescent, executive function, fitness learning, health promotion, interdisciplinary learning, motor ability, physical education, sports skill learning

## Abstract

**Background:**

Previous research has demonstrated that physical exercise enhances motor ability (MA) and that participation in physical activity effectively improves executive function (EF). Physical education (PE), as a core element of students’ daily activities, plays a crucial role in facilitating these benefits. PE also improves the quality of education. Currently, PE are mainly divided into three types. Understanding differences and associations between MA and EF following various PE may improve educational quality, promote physical and mental health. This study aims to: (1) compare the effects of three PE interventions on MA and EF in junior high school students; (2) examine the relationships between MA and EF.

**Methods:**

A cluster randomized controlled trial with a pre-post design was employed. Participants were assigned to Fitness Group (FG; *n* = 21), Sports skill Group (SG; *n* = 20), and Interdisciplinary Group (IG; *n* = 21; sports activities with interdisciplinary knowledge), which calculated value using G*Power. Over 12-week, each group completed twice-weekly interventions. Using BMI, waist-circumference, 20-m-shuttle-run, 30-s-sit-ups, grip-strength, standing-long-jump, 50-m-sprint, sit-and-reach, and 20-s-repeated-crossovers assessed physical fitness (PF). Using questionnaires assessed motor cognition (MC), skill and competition assessed motor skill (MS). MA was evaluated by calculating Z-scores for each component based on age-specific data. EF assessed using the Stroop, 2-back, and More-odd shifting. Paired *t*-tests/Wilcoxon tests, ANOVA/Welch’s ANOVA with *post hoc* comparisons, and Spearman correlations were used for analysis.

**Results:**

MA improved significantly across all groups. ANOVA showed that the specialized sports intervention produced greater improvements in MA, PF, and MC than other groups, with no significant difference in MS. In EF, inhibitory control (IC) and cognitive flexibility (CF) improved significantly, with greater CF gains in the interdisciplinary group (*p* < 0.05) PF was positively correlated with working memory (WM), whereas MC showed a negative association, possibly due to which may reflect the substantial cognitive resources required for complex coordination and motor tasks.

**Conclusion:**

Different PE interventions have distinct effects on adolescents’ MA and EF. The observed associations between MA components and EF domains highlight the close link between physical and cognitive development. These suggest that PE and health promotion policies should integrate structured physical, sports skill, and cognitively engaging interdisciplinary activities to support adolescents’ physical and cognitive development.

## 1 Introduction

Motor ability (MA) refers to the comprehensive capabilities demonstrated during participation in physical activities. In previous research, Stodden et al. define motor competence as the proficiency in fundamental motor skills in children, which influences their participation in physical activities as they age. Children are also able to perceive their own motor competence, and as they grow older, their perception of their abilities becomes more accurate. Furthermore, the study discusses how physical health, particularly health-related physical fitness and obesity, interacts with the relationship between motor competence and physical activity (1). U.S. Department of Health and Human Services defines performance-related fitness, including traits that significantly affect athletic performance, such as aerobic endurance or explosiveness, muscle strength and power, flexibility, speed, and reaction time (2). Sarrazin et al. proposed a multidimensional view of athletic (sport) ability, and in the Conceptions of the Nature of Athletic Ability Questionnaire (CNAAQ), it is mentioned that athletic ability is the product of learning, can be changed, and is specific to certain sports or groups of sports (3). From these studies, it can be seen that MA encompasses a rich content, including physical form, physical fitness, athletic performance, psychological quality, basic athletic ability, and motor skills, emphasizing the comprehensiveness of MA. Therefore, this study adopts the definition provided by Ji in his research, which primarily encompasses three dimensions: physical fitness (PF), motor cognition (MC), and performance of motor skills during competition (MS) (4). PF itself includes body composition, cardiorespiratory endurance, muscular strength, muscular endurance, flexibility, reaction time, displacement speed, coordination, agility, power, and balance. Executive function (EF) constitutes a core component of individual control, memory, and cognitive development. It exhibits a multidimensional structure and represents a complex higher-order cognitive processing system, primarily comprising three sub-functions: inhibitory control (IC), working memory (WM), and cognitive flexibility (CF) (5). EFs regulate and coordinate other cognitive processes to generate coordinated, purposeful behavior (6). They constitute a set of interrelated yet distinct abilities that support beneficial, goal-directed problem-solving capabilities. Physical education (PE) primarily refers to students’ participation in school-based physical activities, serving as a vital component of education. The importance of PE courses in school education is increasingly recognized. Growing research indicates that these courses not only promote students’ physical and mental well-being. Consequently, PE practice courses are no longer confined to PF and specialized sports skills but are gradually evolving toward a diversified development process. Current PE credits are divided into health courses centered on health education and practical PE classes focused on hands-on learning. Based on different teaching content, practical PE classes can be categorized into: (1) Physical fitness instruction, primarily focused on developing physical capabilities; (2) Structured sports skill instruction, centered on learning specific athletic techniques; (3) Interdisciplinary physical education, which incorporates knowledge from other subjects.

Physical education practice has gained widespread recognition for enhancing students’ motor ability. However, existing research primarily focuses on improvements in specific MS or PF. The research can be divided into the impact of specific sports and general sports on the sub-indicators of students’ MA.

Specific sports have been found to enhance students’ motor ability. A study involving children aged 9 to 12 found that after 11 weeks of soccer practice sessions, students demonstrated significant improvements in PF indicators such as body composition compared to a control group, specifically in terms of body fat percentage and lean body mass (*p* < 0.05) (7). One study found that 12 weeks of soccer training reduced fat mass and body fat percentage in 9- to 10-year-old boys (*p* < 0.05) (8). After a 10-week intervention involving three weekly soccer-specific skill classes, junior high students demonstrated significant improvements in their 1,000-m run performance, showing marked differences compared to the control group (9). In a 16-week intervention, it was found that basketball can effectively enhance students’ MS and MC (10).

General sports also play a role in enhancing students’ motor ability. For example, students’ participation in physical activities during PE classes demonstrated superior cardiorespiratory endurance performance compared to daily physical activities (11). When students engage in moderate-to-high intensity physical activities, their muscle strength significantly improves (12). Age 11 represents a sensitive period for children’s speed development (13). Combining 20 min of moderate-intensity motor skill training with 10 min of moderate-intensity physical conditioning effectively reduces 50-m sprint times in elementary and middle school students (14). The engaging nature of PE classes can enhance students’ agility and coordination (15).

Research on motor ability and executive function has shown an increasing trend in recent years. Physical activity may improve cognitive functions in both adolescents and adults, including EF (2). A study of 505 Danish first-grade students found that extracurricular participation in sports significantly improved WM, providing practical support for the correlation between physical activity and children’s WM capacity (16). Research indicates that physical fitness relates to attention resource allocation, cognitive processing, and IC during cognitive processes and stimulus encoding. This was validated using electroencephalography equipment. Magnetic resonance imaging revealed that physical activity effectively influences brain plasticity (17). Numerous studies have supported this viewpoint through controlled trials and reviews. We found that cluster RCTs encompass various types of exercise interventions, interventions utilizing different teaching methods within the same exercise, and interventions involving comprehensive activities. Reviews have also provided evidence.

Some studies have focused on the differences in the impact of interventions involving different types of exercise on students’ executive function. Some researchers demonstrated that aerobic exercise interventions improve children’s EF (18). During a 20-week exercise intervention, students’ EF levels gradually improved over time. Compared to “patterned running,” the “martial arts + jump rope + figure-eight running” combination showed significantly superior IC but inferior WM (19). Research indicates that children participating in team sports demonstrate superior EF performance compared to those engaged in individual sports. Furthermore, children involved in two different types of sports exhibit better EF than those participating in a single sport (20).

There are also studies focusing on the same type of sport, examining the differences in students’ executive function through changes in teaching interventions. Structured tennis instruction demonstrated superior effects on students’ EF compared to single-skill practice sessions following experiential training. Both approaches yielded better executive function performance than learning techniques and matches through televised lessons (21, 22). Studies examining the impact of varying exercise intensities on students’ EF found that moderate-intensity basketball training yielded the most effective intervention for elementary students’ EF (23).

There are also studies focusing on the impact of various activities on students’ physical and mental health, as well as executive function. For instance, some researchers have found that both physical and intellectual activities effectively improved children’s physical health and EF, with combined exercise yielding greater benefits than either modality alone (24). High-frequency, high-intensity physical activities and sports games involving agility and coordination can partially improve children’s EF (25). They noted that PE interventions delivered through a comprehensive curriculum format and increased daily physical activity were more effective for adolescent cognition. It found in an intervention integrating English learning with physical activity that multidisciplinary knowledge integration enhanced students’ EF levels (26). They found the enriched PE program (interventions incorporating more cognitive challenge tasks and teaching methods) beneficial for the inhibitory component of cognitive executive function, but not for working memory updating or attention, suggesting differential associations between specific aspects of motor competence and specific executive processes, and found that create Physical Activity intervention programs in PE that stimulate children’s EF and provide evidence on the beneficial effects of thoughtful experiences on the developing brain (27). Engaging in sports with high cognitive involvement may significantly enhance an individual’s EF (28). These studies provide a foundation for the current research to focus on the impact of interdisciplinary learning on students’ cognitive function.

Reviews also provide evidence for the impact of physical activities on executive function. A meta-analysis of literature on EF and physical activity revealed substantial effect sizes for exercise on WM, IC, and CF. This indicates exercise effectively enhances EF across multiple stages of child development (29). In a systematic review incorporating 25 randomized controlled trials, they found that PE positively impacts cognitive functions in elementary school students, primarily manifesting as improvements in attention, creativity, memory, academic performance, and IC (30). In a meta-analysis incorporating 36 studies, they found an effect size of 0.20 (95% CI = 0.10–0.30) for EF (31).

Studies indicate that motor ability are not only crucial for physical and mental health but also significantly correlate with cognitive abilities and executive function. Additionally, they can influence the activation levels of brain regions associated with higher-order cognition (32). Some studies have demonstrated a relationship between MA and EF. Some cross-sectional studies provide evidence suggesting that motor ability may be related to executive function. Testing 131 students revealed significant correlations between physical exercise (touch test pad, agility, lower and upper limb explosive strength) and EF (33). The subtle yet critical role of EF in motor decision-making (34). Some people found that enhancing children’s gross motor competence may improve PF (cardiorespiratory fitness, speed/agility, upper and lower body strength) and cognitive abilities (IC, WM, CF) (35). Additionally, studies have predicted adolescent tennis competition outcomes based on EF levels (21, 22). Some researchers found that composite measures of exercise-related PF were the strongest predictors of EF (33).

Through findings from relevant research, it is evident that current studies on physical activity and executive function are quite extensive, but they mainly focus on one aspect, such as physical fitness, motor skill, or motor cognition performance. Research on the intervention effects of sports on executive function has primarily focused on traditional PE instruction, lacking a comparison of the intervention effects on interdisciplinary learning and the differences in intervention effects on physical fitness and specific sports skills. Most studies exploring the relationship between MA and EF are cross-sectional studies. There is a lack of cluster RCTs examining the correlation between MA and EF after PE intervention. In addition, we have found that research on the correlation between different types of physical education and health courses and students’ MA and EF is still insufficient. By focusing on this relationship, it would provide experimental evidence for the impact of different types PE on students’ MA and EF. The study selected junior high school students as the research group, as children at this stage experience rapid physical and cognitive development, and are capable of engaging in more complex motor and cognitive activities. The results of the study will help guide the development of school sports programs and assist PE teachers in designing effective PE courses, thereby improving students’ MA and EF development, enhance physical and mental health.

Therefore, the main objective of this study is: (1) to determine whether three different types of physical education courses (Physical fitness training, Sports skill learning and training, and Learning and practicing sports activities with interdisciplinary knowledge) have different impacts on students’ MA and EF, (2) to explore the correlation between MA and EF.

## 2 Methods

### 2.1 Participants

Sample size for this study was calculated using G*Power (version 3.1.9). Based on prior research (supplementary literature), an effect size of 0.25, a significance level of 0.05, and a power of 0.80 were specified. With 3 groups, 2 measurements, and an assumed correlation coefficient of 0.5, the minimum sample size required was determined. Calculations yielded a minimum sample size of 18 participants per group, totaling 54 participants. Accounting for attrition, early withdrawal, and sports injuries, a total of 62 participants were enrolled. All participants were junior high school students in the same grade level, comprising 31 males (50.0%) and 31 females (50.0%).

The participant random sampling process was as follows:

A total of six classes (n = 271 students) were recruited in this study. Each class was assigned a numerical code (Classes 1–6). Within each class, students were coded by gender (male = 1, female = 2) and ordered alphabetically by surname.

Stratified random sampling by sex was conducted within each class to ensure gender balance. The numbering format was Class-Gender-Number. For example, a female student in Class 1, sorted alphabetically by surname as number 16 within the class, would be numbered as 1-2-16. The participants were then randomly selected on the computer, ensuring that the sample size and gender balance were maintained as closely as possible to meet the overall sample requirements. Specifically, 10–11 students were randomly selected from each class while maintaining an approximately equal number of males and females: 10 students from Class 1 (5 males, 5 females), 11 from Class 2 (6 males, 5 females), 10 from Class 3 (5 males, 5 females), 11 from Class 4 (6 males, 5 females), 10 from Class 5 (5 males, 5 females), and 11 from Class 6 (5 males, 6 females). A total of 63 participants were ultimately included.

Following participant selection, Classes 1 and 2 were combined to form Cluster A, Classes 3 and 4 formed Cluster B, and Classes 5 and 6 formed Cluster C. Cluster-level randomization was then performed. Three identical slips of paper labeled “FG,” “SG” and “IG” were placed into opaque envelopes. One representative from each cluster (A, B, and C) drew one envelope under the supervision of 3 teachers who were not involved in outcome assessment or intervention delivery.

The cluster that drew “FG” was assigned to the Fitness Training Group (*n* = 21), the cluster that drew “SG” was assigned to the Specific Sport Skills Instruction Group (*n* = 20), and the cluster that drew “IG” was assigned to the Interdisciplinary Learning Instruction Group (*n* = 21).

One participant was excluded due to failing to achieve the required accuracy threshold of 0.80 on the executive function tasks. A total of 62 participants were ultimately included in the study. All 3 groups comprised students with no significant differences in MA and EF performance. Participants’ basic demographic information is presented in Table 1.

**Table 1:** Basic demographic characteristics of participants.

Demographic variables (*n* = 62)							
Variable	Category	Male		Female		Total	
		*f*	%	*f*	%	*f*	%
Intervention method	Fitness training	11	52.3	10	47.6	21	33.8
	Sports skill learning and training	10	50.0	10	50.0	20	32.2
	Interdisciplinary learning	10	47.6	11	52.3	21	33.8
Total		31	50.0	31	50.0	62	100.0

A convenience sample of 62 junior high school students was recruited. Participants learned about the experiment through teacher explanations and voluntarily enrolled via classroom recruitment notices. Interested students provided personal details including name, class, age, and parental contact information. Eligible individuals were invited to participate after obtaining parental or guardian consent. Recruitment continued until the target sample size was reached.

During recruitment, participants were screened for prior medical history, family history of cardiovascular disease, and physical conditions to exclude potential exercise risks. Additional criteria included no injury history within the month preceding the study, no medication use, no special educational needs.

This study was approved by the University Committee on Human Research Protection at East China Normal University (Approval No.: HR667-2024). All participants and their parents or primary guardians were informed about the experimental procedures and potential risks, and voluntarily signed informed consent forms. No subjects withdrew from the study, and no financial incentives were provided.

### 2.2 Design and procedure

A cluster randomized controlled trial with a pre–post design was employed. Three test administrators conducted all measurements without participating in the intervention. The result evaluators were unaware of the group allocation during the measurement and analysis. The subjects were numbered in sequential order. Pre-tests commenced at the semester’s start, beginning November 15th and lasting 1 week. Testing included demographic information, measures of MA (PF, MC, MS), and assessments of EF (IC, WM, and CF). Participants received a 12-week intervention starting November 18th, comprising physical fitness training, soccer-specific motor skills, and interdisciplinary learning lessons. Post-tests were administered upon intervention completion. To account for extraneous variables potentially affecting MA and EF performance, participants reported their daily physical activity levels.

On test days, participants refrained from vigorous physical activity beyond routine exercise and the experimental intervention for 12 h prior. They also avoided caffeinated beverages and maintained consistent dietary patterns during the testing phase to minimize baseline variability, ensuring pre-test results were unaffected by dietary nutrition or additional physical exertion.

During the pre-test, participants first completed demographic information registration. Subsequently, after sitting quietly for 6 min in a dimly lit, quiet classroom, participants began the executive function task testing. Participants were positioned 30–60 cm from the computer screen, adjusted according to their vision and height. To minimize testing variations from extraneous variables, each participant completed practice runs following task-specific instructions before starting. All EF tasks were administered in a consistent order. First, participants completed the Stroop task to assess inhibitory control. Second, participants completed the N-back task to measure working memory. Finally, they performed the MOS (More-Odd Shifting) task to test their shifting abilities. A one-minute rest period separated each task. The testing computer automatically recorded data throughout the process.

Motor ability measurements followed. PF comprised body composition, physical endurance, motor cognition, and motor skill performance. (1) Participants underwent body composition testing for height, weight, and waist circumference. (2) After a 5-min warm-up, participants completed physical endurance tests including 20-m shuttle runs, 50-m sprints, grip strength, sit-ups, standing long jump, sit-and-reach, and 20-s repeated crossovers. (3) Participants underwent MS assessments. (4) Participants also completed a MC evaluation questionnaire. Test administrators recorded PF data, compiled and documented questionnaire results, and conducted further analysis.

The post-test followed the same procedure as the pre-test, beginning with EF tasks followed by MA assessments. Testing was scheduled in the morning or afternoon based on participants’ availability.

### 2.3 Intervention

The intervention period is expected to last 12 weeks. During this time, participants in each experimental group will attend classes taught by uniformly trained physical education teachers. Each experimental group will participate in two 40-min physical education classes per week. The content of the teaching experiments varies by experimental group:

The Fitness Group received the following instruction: (1) A 7-min warm-up and preparation phase, comprising a 2-min classroom routine and a 5-min warm-up; (2) The main segment featured 28 min of physical fitness activities, completing 4 different types of fitness exercises per session (e.g., body composition, muscular strength, muscular endurance, cardiorespiratory endurance, flexibility, reaction ability, displacement speed, coordination, agility, explosive power, balance, etc.), with each exercise lasting approximately 5 min. This segment totals 20 min, followed by an 8-min physical activity competition or demonstration; (3) The closing segment includes 5 min of relaxation activities and a class summary: 4 min for relaxation activities and 1 min for the class summary and teacher feedback.

The instructional content for the Sports Skill Group as follows: (1) The warm-up and preparation segment consists of a 7-min classroom routine and warm-up activities, including a 2-min classroom routine and a 5-min warm-up; (2) The main segment featured 30 min of soccer activities: 4 min for learning and practicing individual and combination soccer techniques; 8 min for two or more games or mini-matches using individual and combination techniques; 8 min for small-sided soccer matches; and 10 min for diverse, compensatory, fun, and integrated physical exercises; (3) The closing segment includes 5 min of cool-down activities and class summary: 4 min for cool-down activities and 1 min for class summary and teacher feedback.

The Interdisciplinary Group receives the following instructional content: (1) The beginning and preparation segment includes 7 min for classroom routines, warm-ups, and contextual introduction, comprising 2 min for classroom routines and 5 min for warm-ups and contextual introduction; (2) The main segment features 30 min of sports games, competitions, or demonstrations centered around a theme and integrating multiple disciplines; (3) The closing segment includes 5 min of relaxation activities and a class summary: 4 min for relaxation activities and 1 min for the class summary and teacher feedback. The following presents a segment of the teaching case.

After warm-up activities, students will be divided into groups for a 10-min competition. During the competition, performance indicators were systematically recorded, including total passes, successful passes, shots attempted, and goals scored. Based on these data, passing success rate, shooting accuracy, and average number of ball touches per player were calculated. Discussion: Does a high success rate necessarily mean victory? How can data help improve collaboration? This part takes about 5 min.

Subsequently, a shooting experiment was conducted to examine the relationship between movement patterns and ball displacement. Students performed both stationary shooting and run-up shooting. Students waiting in line perform stationary lunge jumps. This part takes about 8 min. The rolling distance of the ball, total shots, and goals scored were recorded, and shooting accuracy was calculated. This part takes about 3 min. Discussion: Why does the rolling distance of the ball differ when shooting with a running run compared to shooting from the ground? Why is there such a result? What insights have they provided for us in football and competitions? This part takes about 4 min.

Throughout the intervention, instructors strictly adhere to the teaching plan to prevent participant injury and ensure their well-being. In the first class of the intervention, the subjects were equipped with Polar watches to control the average exercise intensity of the class within the range of 140 beats per minute to 160 beats per minute. Participants should immediately report to instructors if they feel the exercise intensity is too high, or if they experience any discomfort or potential injury, so they can rest and receive treatment as needed. If the teaching plan is interrupted due to inclement weather conditions or holidays, indoor sessions focusing on exercise theory knowledge will be conducted during inclement weather. Instructors document participant status throughout the entire intervention.

### 2.4 Measurement

#### 2.4.1 Motor ability

##### 2.4.1.1 Physical fitness

Physical fitness testing includes BMI, waist circumference, 20-m shuttle run, 30-s sit-ups, grip strength, standing long jump, 50-m sprint, sit-and-reach, and 20-s shuttle run (36).

1 Height

Students stand barefoot with heels together on the measuring device, keeping the torso naturally upright. The height measurement device displays the subject’s height. Measurements are taken twice, recorded by the researcher on the questionnaire form to the nearest hundredth of a centimeter (cm).

2 Weight

The student stands naturally on the weighing scale, maintaining a stable posture for 3 s. The researcher reads the data, performs two measurements, and records the results in the questionnaire form, accurate to one decimal place (0.01) and expressed in kilograms (kg).

3 Waist circumference

Researchers wrap a tape measure horizontally around the student’s waist, positioned 1 cm above the navel. The student stands naturally and maintains steady breathing. When reading the measurement, researchers ensure their line of sight is level with the scale. Measurements are taken twice, recorded in the questionnaire form, and reported to the nearest hundredth (0.01) in centimeters (cm).

4 20-m shuttle run

Students performed thorough warm-up and stretching exercises and watched instructional videos. The entire test followed musical rhythms, assessing CRF endurance through a 20-m shuttle run. Participants moved from one line to another 20 m away, then reversed direction based on audio signals indicating speed. The initial speed is 8.5 km/h, increasing in 0.5 km/h increments per minute (1 min equals one stage). The test ends when the participant fails to reach the finish line in sync with the audio signal twice consecutively. Otherwise, the test concludes when the participant stops due to fatigue. The last completed stage or half-stage before withdrawal is scored.

5 50-m sprint

Divide students into groups of five. Use a standing start. Two researchers conduct the test: one positioned beside the starting line to signal the start and time the run, while the other, positioned at the starting point, begins timing upon flag signal and stops the timer when the student’s chest crosses the finish line. Record and enter the result in the questionnaire table, accurate to the hundredth decimal place (0.01).

6 30-s sit-ups

Students lie flat on a gymnastics mat with arms crossed over the chest, hands resting on shoulders, knees bent at 90 degrees, with feet flat on the ground. Students engage their core muscles to lift their upper body until both elbows touch the knees, completing one repetition. Avoid holding breath or placing hands behind the head during the exercise. Perform continuously for 30 s. Record the result to the nearest whole number (1.0) in the questionnaire table, using the number of repetitions (n) as the unit.

7 Grip strength

Stand naturally. Grasp the HUWAIREN grip strength tester. The researcher adjusts the grip width based on the student’s gender and age. The other hand rests naturally at the side. During the test, the student must exert force rapidly. Perform two tests. Record and enter the results in the questionnaire table, accurate to one decimal place (0.01), using kilograms (kg) as the unit.

8 Standing long jump

Students stand with feet shoulder-width apart, toes positioned behind the starting line. Upon the researcher’s signal, students jump vertically in place without taking a running start. The jump must not result in overstepping the line due to a hop or double-step. The test distance is measured from the starting line to the closest point of the student’s body upon landing. Two attempts are recorded, with results entered into the questionnaire table to the nearest hundredth (0.01) in centimeters (cm).

9 20-s repeated side-to-side stepping

Before the test begins, play a demonstration video and key points, and demonstrate to the testers as follows: Students stand with feet apart on the middle line of three parallel lines spaced 1 m apart. Upon a whistle signal, students collectively begin by side-stepping from left to right over the rightmost line. They proceed sequentially across all three parallel lines. The test ends with another whistle signal, and results are recorded on the questionnaire form. Crossing one parallel line counts as one repetition. Precision to one decimal place (1.0), unit: number of times (n).

10 Sit-and-reach test

Students sit upright on a flat mat with legs extended straight in front of the apparatus. Arms are naturally extended downward, palms facing down, with both hands placed on the apparatus’s flat plate. Students slowly push the apparatus’s stop plate forward with both arms until the waist tilts forward to the point where further forward movement is impossible. Upon test completion, the device displays results. Record measurements on the questionnaire form to the nearest hundredth (0.01) in centimeters (cm).

##### 2.4.1.2 Motor cognition

The motor ability Scale for Junior High School Students developed by Dai Shengting was used to assess participants’ MC levels (37). This 8-item scale employs a 5-point Likert scale with no reverse-scored items. The standardized Cronbach’s *α* coefficient for this version is 0.916. Research data reliability coefficients exceeding 0.9 indicate high data reliability and good homogeneity, confirming this as a highly reliable scale that effectively reflects junior high students’ MC levels. In the current study, Cronbach’s Alpha reliability coefficient was found to be 0.967. This value demonstrates the scale’s high reliability for this research. To provide a clearer overview of the measurement tools used in this study, the items, subdimensions, and indicators of the MC Scale are summarized in the Supplementary Table 1. Motor Cognition Scale - Item Summary.

##### 2.4.1.3 Motor skills

Technical Specifications: Starting 2 m from the penalty area line, place 8 markers along a 20-m vertical line, each spaced 2 m apart. The 8th marker is positioned 4 m from the starting point. The starting line is 4 m long, perpendicular to and intersecting the 20-m line. Test Method: Upon hearing the start signal, the subject dribbles the ball continuously around all eight markers. Before the ball rolls into the penalty spot, males must shoot using the instep, while females may use either the instep or the ball of the foot. The stopwatch is stopped when the entire ball crosses the goal line vertically between the goalposts and under the crossbar. Any of the following situations constitutes a violation of test regulations, resulting in the invalidation of that attempt without additional attempts: the subject fails to dribble past a marker pole, fails to shoot before the ball reaches the penalty spot, uses the toe to poke the ball into the goal, or the ball fails to enter the goal. If the ball hits the crossbar or goalposts and rebounds into play, one retest is permitted. Each participant receives two attempts, with the best result recorded.

Competition: Matches are held every other Wednesday afternoon, lasting 20 min each, with a total of 5 matches. Scoring Criteria: Evaluators independently assess candidates’ technical ability, tactical awareness, mental resilience, and competitive conduct based on practical performance guidelines. Goals scored during matches serve only as one reference point for scoring, not the primary criterion. Three evaluators will score according to a unified standard. Goals will only serve as a reference point for scoring, not the main scoring criterion. To ensure consistency in scoring, all evaluators will undergo standardized training before the competition to ensure they understand and accurately apply the scoring criteria. Scoring consistency: To ensure the fairness of the scoring, after each game, the evaluators’ scores will be reviewed by a third party. If there are significant differences in scoring, the final scores will be discussed and adjusted to ensure that each student receives a fair evaluation. Scoring uses a 10-point scale, with scores rounded to one decimal place.

Assessment Rubric is in Supplementary Table 2. Motor Skills Scale–Item Summary.

#### 2.4.2 Executive function

The executive function task paradigm employed in this study comprises three task paradigms: Inhibitory Control (Stroop task paradigm), updating function and Working Memory (2-back task paradigm), and Cognitive Flexibility (More-odd shifting task paradigm). The EF assessment required participants to operate using the E-prime 3.0 software system on a computer. A correct response rate of 0.8 or higher was required for the EF tasks. Only one participant did not meet the 0.8 threshold and was therefore excluded, while the remaining participants met this requirement. The final number of participants was 62. Executive function levels were jointly represented by reaction time (RT) and accuracy (ACC). Shorter RT and higher ACC indicate higher levels of each EF subcomponent. IC RT is estimated using the difference between incongruent and congruent RT. WM RT is assessed using 2-back RT. CF RT is estimated using the difference between shifting and non-shifting RT. The specific procedures for assessing IC, EM, and CF in adolescents aged 13–18 are as follows.

##### 2.4.2.1 Inhibitory control—Stroop experimental paradigm

**Figure 1:**
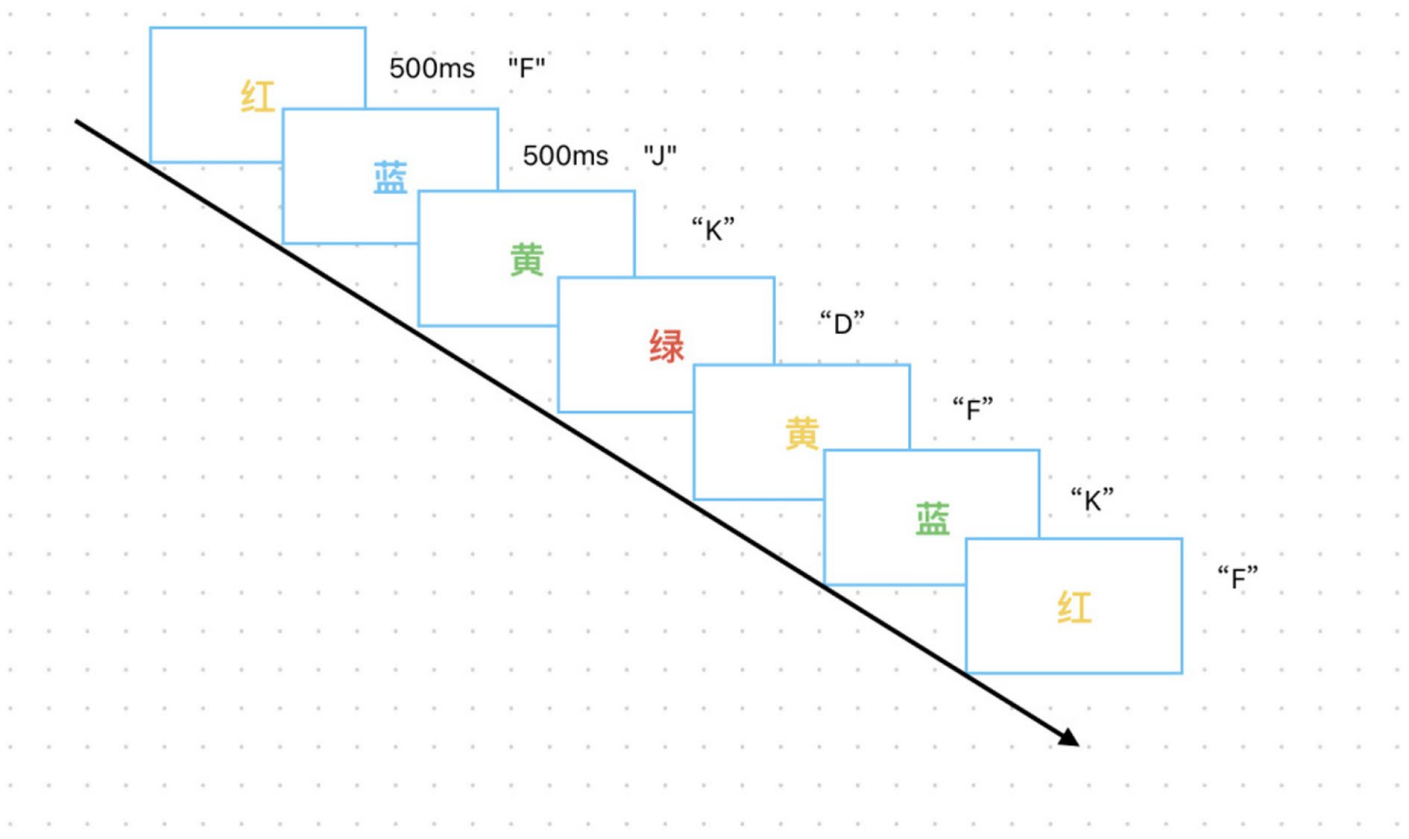
This figure shows the task flow of the Stroop experiment.

The experimental program was developed using E-prime 3.0. Each task comprised 4 blocks, with each block containing 30 trials. During testing, a colored Chinese character appeared randomly on the screen in front of the subject, who was required to identify the color of the character. The experimental materials consisted of four Chinese characters: “红” (red), “黄” (yellow), “蓝” (blue), and “绿” (green). The color of each character appeared randomly as “红,” “黄,” “蓝,” or “绿.” The colors appear with equal probability (1:1:1:1) and are randomly presented during testing. At the start of the test, a target “+” symbol appears on the screen, followed by the stimulus material. The participant then makes a judgment and responds by pressing a key. Participants are instructed to make judgments as quickly and accurately as possible. The “+” symbol reappears on the screen afterward, and the cycle repeats. In the Stroop color-word task, subjects judge the color of the Chinese character. If the character is red, press “D”; if yellow, press “F”; if blue, press “J”; if green, press “K.” After pressing the key, the next colored character appears. The entire test lasts approximately 5 min. See Figure 1 for the specific task flow.

##### 2.4.2.2 Working memory—2-back experimental paradigm

**Figure 2:**
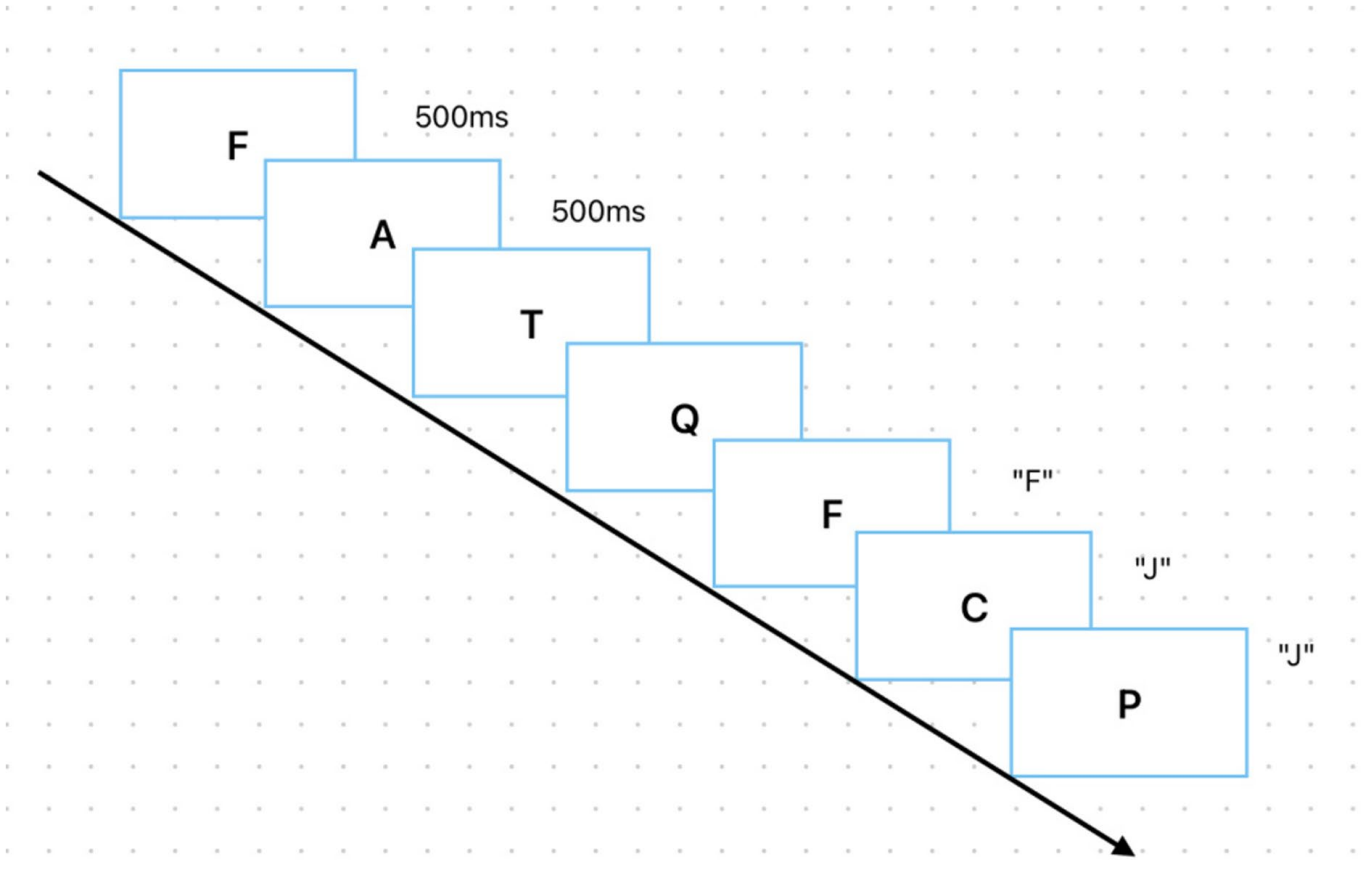
This figure shows the task flow of the 2-back experiment.

The second behavioral experiment employed a spatial n-back task programmed using E-prime 3.0. This task comprised four blocks, each containing 30 trials. Alphabet letters served as stimuli, randomly presented on the participant’s screen. At the start of testing, a target “+” symbol first appeared on the screen, followed immediately by the stimulus material. Participants were required to rapidly process the information during the blank screen period and respond by pressing a key. The black screen period served as the response time window. Participants were required to provide rapid responses to the stimuli within a maximum window of 500 ms, with the cycle repeating. During the 2-back task, participants compared the second letter in a sequence with the third letter. If the letters matched, they pressed the “F” key; if they differed, they pressed the “J” key. The entire n-back task test lasts approximately 5 min, with a 5-s interval between each block for rest. The specific task flow is shown in Figure 2.

##### 2.4.2.3 Cognitive flexibility—MOS (more-odd shifting) task

**Figure 3:**
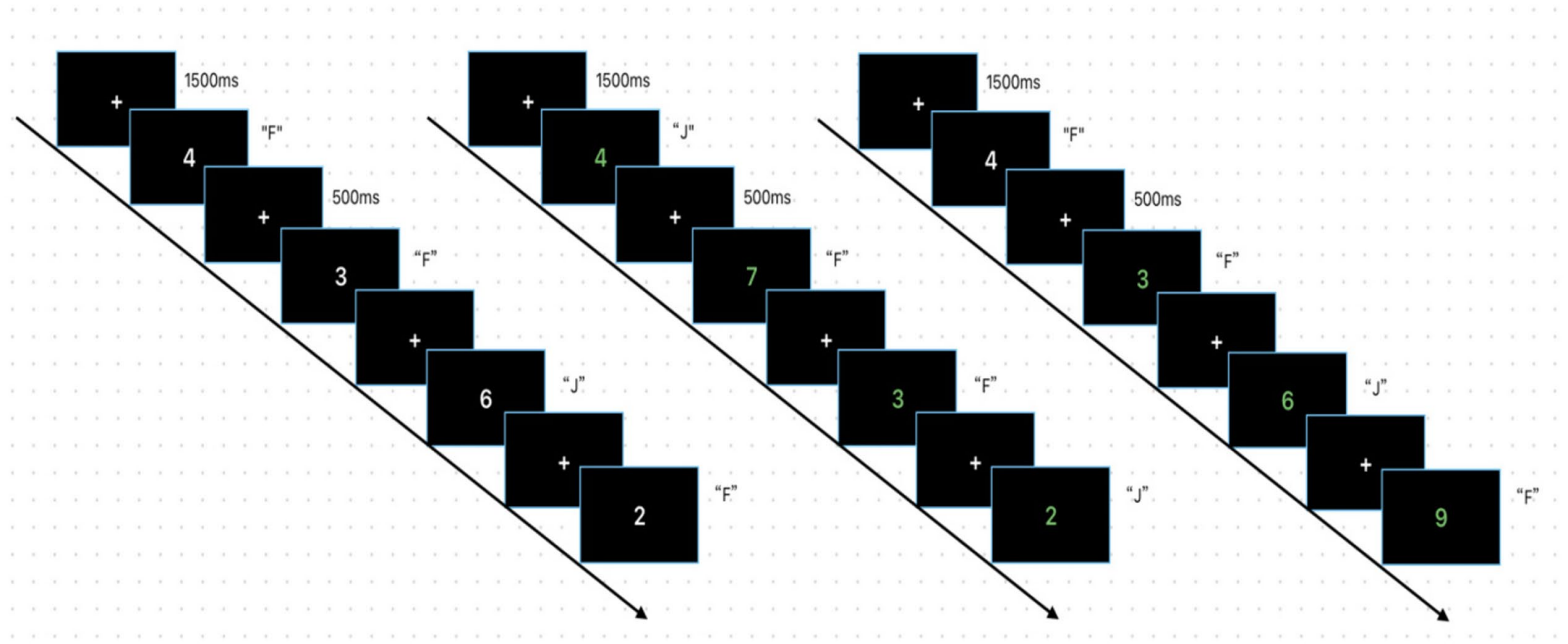
This figure shows the task flow of the MOS experiment.

The third behavioral experiment employed a spatial MOS task programmed using E-prime 3.0. Each task comprised two blocks, with each block containing 15 trials. Stimuli consisted of the Arabic numerals 1, 2, 3, 4, 5, 6, 7, 8, and 9, presented in either white or green coloration. These stimuli appeared randomly on the screen in front of the participant. The task comprised three components: a digit magnitude judgment task (white digits), an odd-even digit task (green digits), and a mixed magnitude-odd-even task (randomly presenting white or green digits). The task began with a 1,500 ms target “+” symbol displayed centrally on the screen, followed by the presentation of the stimulus material until the participant made a judgment and responded via key press. In the magnitude judgment task, when a white digit appears, the participant compares it to “5”: press “F” if smaller than “5,” press “J” if larger than “5.” “5” does not appear. Then a 500 ms target “+” appears in the center of the screen, and the cycle repeats. In the odd/even judgment task, when a green number appears, the participant must determine its odd/even nature: press “F” for odd, “J” for even. A 500 ms target “+” then appears in the center of the screen, and the cycle repeats. In the size-odd/even judgment task, when a white number appears, the participant must judge its size: press “F” if it is less than “5,” “J” if it is greater than “5.” The number “5” does not appear. When a green number appears, the subject must determine its parity: press “F” for odd numbers and “J” for even numbers. After a 500 ms target “+” mark, the cycle repeats. The total task duration is approximately 4 min. See Figure 3 for the specific task flow.

### 2.5 Statistical analysis

Enter the collected data into Excel for preliminary analysis, including the removal of outliers.

#### 2.5.1 Motor ability

When analyzing data related to motor ability, which encompasses physical fitness, motor skill and motor cognition, data sources included health tests, questionnaires, skill assessments, and competitions. Due to inconsistencies in measurement units across components, *Z*-scores were employed for data analysis. The specific calculation method is as follows:

##### 2.5.1.1 Calculation of physical fitness data

This study estimated maximum oxygen uptake VO₂max using the 20-m shuttle run test to represent participants’ cardiorespiratory endurance. The evaluation method employed the VO₂max prediction equation for Chinese adolescents aged 13–18 years developed, and it has already been validated in China (38). The specific formula is as follows:


$$\begin{array}{l} {\mathrm{VO}}_{\mathrm{2max}}=62.103+(0.302\;\text{laps})–(0.877\times \mathrm{BMI})\\ –(4.874\times \text{gender})–(0.465\times \mathrm{age})\\ \;\;\;\;\;\;\;\;\;\;\;\;\;\;\;\;\;\;\;(\begin{array}{l} \text{laps}=\text{number of}\;20m\;\mathrm{SRT}\;\text{repetitions};\\ \text{male}=0;\text{female}=1;R^{2}=0.522 \end{array}) \end{array}$$

Standardize BMI, waist circumference, VO_2max_, grip strength, 30-s sit-ups, standing long jump, 50-m sprint, sit-and-reach, and 20-s repeated crossovers (Z-scores), and calculate corresponding *Z*-scores. Among these, VO_2max_, grip strength, 30-s sit-ups, standing long jump, 50-m dash, sit-and-reach, and 20-s shuttle run were calculated using the mean and standard deviation for each gender and age group referenced from the “Physical Fitness Evaluation for Chinese Children and Adolescents” (36). The formula is as follows, where M is the mean value for the respective item and SD is the standard deviation for that item.


$$Z=\frac{X-M}{\mathrm{SD}}$$

In calculating the *Z*-score for each physical fitness item, the 50-m run value represents the subject’s speed, where shorter times indicate better performance. Therefore, negative *Z*-scores are used for this item. The *Z*-score for the 50-m sprint is inverted, and all *Z*-scores are summed to obtain the PF. The formula is as follows:


$$\begin{array}{l} \mathrm{PF}=Z_{\mathrm{VO}2\max}+Z_{30\text{sSit}-\mathrm{ups}}+Z_{\text{Grip Strength}}\\ +Z_{\text{Standing Long Jump}}+Z_{\mathrm{Sit}-\text{and}-\text{Reach}}+Z_{20\text{sShuttle}\;\mathrm{Run}}\\ -Z_{50m\;\text{Sprint}}-Z_{\mathrm{BMI}}-Z_{\text{Waist Circumference}} \end{array}$$

##### 2.5.1.2 Calculation of motor skill data

According to the requirements of China’s National Physical Fitness Testing Standards, skill assessment scores are converted to a percentage scale with a total of 100 points. To facilitate the calculation of athletic skill data, student skill assessment scores are weighted at 50%, i.e., skill assessment score multiplied by 50%. Records are kept for the soccer matches held every Wednesday afternoon, totaling 5 matches. Winning team members may earn up to 10 points per match based on their on-field performance. Members of teams that draw may earn up to 8 points per match based on their on-field performance. Members of losing teams may earn up to 7 points per match based on their on-field performance. Finally, the class PE teacher transcribes and records these scores. The cumulative score from these five matches constitutes the student’s MS score. Students who frequently miss matches due to reasons receive a maximum of 20 match points. Students unable to participate due to illness but assisting with scoring receive 5 match points per game. Thus, MS performance = skill assessment score × 50% + cumulative match score. This study assessed students’ MS performance through a 20-m soccer dribbling-and-shooting combination skill test and soccer matches. Scores were converted to a percentage-based evaluation system aligned with China’s National Physical Fitness and Health Testing Standards. To control data variability, *Z*-scores were calculated using the mean and standard deviation of MS scores from all students in the same grade at the experimental school.

##### 2.5.1.3 Calculation of motor cognition data

The motor cognition questionnaire employed in this study was the “Motor Cognition Evaluation Questionnaire for Junior High School Students” developed by Chinese scholars. Following its development, the questionnaire was administered to students in the Yangtze River Delta region, yielding 4,058 valid responses. The regions and educational stages covered by this questionnaire align with the distribution of subjects in this study. Therefore, the calculation of *Z*-scores in this research utilizes the mean and standard deviation from the study “Questionnaire” by Chinese scholars (37).

**Calculation of motor ability data**


$$\mathrm{MA}=\mathrm{PF}+Z_{\mathrm{MC}}+Z_{\mathrm{MS}}$$

#### 2.5.2 Executive function

Collect data using E-prime 3.0 and assign identification numbers. Before testing began, experimenters explained the purpose and significance of the test, outlined procedural rules and precautions, demonstrated the program’s operation flow, and addressed student inquiries to ensure all junior high students genuinely understood the requirement to improve key response speed while maintaining accuracy. Each student was informed of their assigned identification number. As this study involved two measurements, numbers were verified with students before each session to ensure data validity. Students were instructed to answer carefully and attentively throughout testing. After testing, experimenters compiled all data, copying ACC and RT from each task into an Excel spreadsheet for further processing. They calculated ACC and RT for the Stroop task, 2-back task, and MOS task. After aggregating all participants’ ACC and RT data, to examine the effects of different course types on students’ executive functions.

#### 2.5.3 Effect analysis

After preliminary data calculation and analysis, for the three groups (FG, SG, IG) and two time points (pre-test and post-test) to examine Normality plots with Shapiro–Wilk tests and examine Levene Test. When *p* > 0.05, the assumptions of normality or homogeneity of variance were considered satisfied. Paired-samples t tests were conducted for data meeting the normality assumption. For data satisfying the homogeneity of variance assumption, two-way ANOVA, ANCOVA, and LSD *post hoc* multiple comparisons were performed. When the normality assumption was violated, the Wilcoxon signed-rank test was applied. When homogeneity of variance was not met, Welch’s ANOVA and Games–Howell post hoc test multiple comparisons were used. These analyses were conducted to examine the effects of different conditions and within-condition changes on MA and EF.

For analysis of variance, effect sizes are expressed as partial eta squared (η^2^). For post comparisons, effect sizes are expressed as partial eta squared (η^2^). The significance level was set at 0.05. Effect sizes of η^2^ and d were classified as (0.01, 0.06, 0.14), (0.2, 0.5, 0.8). Outliers were identified using standardized *z*-scores (|*z*| > 3). Additionally, analyses were repeated after excluding potential outliers, and the results remained consistent. Statistical analyses were conducted using SPSS (Version 29, IBM SPSS, Armonk, NY, USA).

#### 2.5.4 Correlation analysis

After pooling the ACC and RT data from all participants across the three EF tasks, correlations were calculated between these measures and the standardized *Z*-scores of the overall MA score and its subcomponents (PF, MC, MS). This explored the relationship between MA and EF. Spearman’s correlation analysis was used to examine the pairwise associations between MA and its components with EF post-intervention. Spearman’s correlation analysis was used to examine the pairwise associations between MA and its components with EF post-intervention. Spearman’s correlation was chosen due to the non-parametric nature of the data, as the normality assumption was not met. The Shapiro–Wilk test was conducted to assess the normality of the data, and the results indicated that some data were non-normally distributed. Therefore, non-parametric Spearman’s correlation was applied to better capture the monotonic relationships between the variables.

## 3 Results

Participants’ demographic characteristics showed relatively balanced distribution across experimental groups and genders. Specifically: (1) 21 participants (33.8%) engaged in FG, comprising 11 males (52.3%) and 10 females (47.6%). (2) Twenty participants (32.2%) enrolled in SG, comprising 10 males (50.0%) and 10 females (50.0%). (3) Twenty-one participants (33.8%) enrolled in IG, comprising 10 males (47.6%) and 11 females (52.3%) (Table 1).

### 3.1 Motor ability results

First, a paired *t*-test was used to analyze differences in motor ability across experimental groups before and after intervention. Subsequently, a covariance analysis was conducted with pre-test scores as covariates to determine whether significant differences existed between groups after controlling for pre-test levels, thereby assessing how different PE courses influenced MA in junior high students. Subsequently, a covariance analysis was conducted for different genders to examine whether the effects of different intervention methods on students’ MA varied by gender. Finally, a two-way ANOVA was performed to test the interaction effect of intervention method × gender on students’ MA. The results of these analyses, along with relevant tables and charts, are presented below.

Upon examining the results, the main effect of group was significant (*F* = 4.391, *p* = 0.017, η^2^ = 0.138), indicating meaningful differences among the experimental groups. Conversely, no significant differences were observed in the MA variable across genders (*p* > 0.05). Furthermore, no significant interaction effect was found between teaching intervention method and gender on the MA variable (*p* > 0.05). As shown in Table 2.

**Table 2:** Main effects of different instructional methods and gender on motor ability after controlling for pre-test consistency.

Source	Type III sum of square	df	MS	F	p	η2
Corrected model	504.436a	6	84.073	7.342	<0.001	0.445
Intercept	809.402	1	809.402	70.684	<0.001	0.562
Pre-motor ability	358.797	1	358.797	31.333	<0.001	0.363
Group	100.572	2	50.286	4.391	0.017	0.138
Gender	31.102	1	31.102	2.716	0.105	0.047
Group * Gender	42.596	2	21.298	1.860	0.165	0.063
Error	629.801	55	11.451			
Total	6893.186	62				
Adj. total	1134.237	61				

df, Degrees of Freedom; MS, Mean Square.

The results indicate that significant differences were observed between groups in MA, with the SG showing the most pronounced improvement. However, no significant gender × intervention interaction effect was found, indicating that the effect of the SG intervention did not differ significantly between males and females. Although the simple main effect of group was significant among female students, and descriptive statistics indicated relatively greater gains for females in the SG group, these differences should be interpreted as within-group effects or descriptive trends rather than statistically significant gender-specific effects. Therefore, the findings support the overall effectiveness of the SG intervention in improving MA, but do not provide evidence that it was more effective for one gender than the other. As shown in Tables 2, 3.

**Table 3:** Mixed-effects analysis comparing the effects of different instructional methods and gender on motor abilities (physical fitness, motor skills, motor cognition) after controlling for pretest consistency.

**Group**	**Variable**	**Male** **(*n* = 31)**			**Female** **(*n* = 31)**			**Total** **(*n* = 62)**		
		**FG** **(*n* = 11)**	**SG** **(*n* = 10)**	**IG** **(*n* = 10)**	**FG** **(*n* = 10)**	**SG** **(*n* = 11)**	**IG** **(*n* = 10)**	**FG** **(*n* = 21)**	**SG** **(*n* = 21)**	**IG** **(*n* = 20)**
Motor ability	Pre: Mean (SD)	5.74 (5.28)	4.34 (4.44)	7.05 (4.31)	4.51 (4.2)	7.77 (4.54)	6.56 (3.86)	5.15 (4.72)	6.14 (4.72)	6.8 (3.99)
	Post: Mean (SD)	8.53 (6.30)*	11.69 (4.00)*	10.33 (3.86)*	8.77 (3.78)	11.14 (3.34)*	7.34 (2.87)ab*	8.64 (5.13)*	11.4 (3.58)a*	8.83 (3.65)b*
		Group			Group			Group	Gender	
	*F*	1.11			5.80			4.00	2.02	
	*p*	0.35			0.01#			0.02#	0.16	
	η^2^	0.08			0.30			0.12	0.03	
Physical fitness	Pre: Mean (SD)	7.02 (5.48)	4.57 (4.78)	7.25 (2.87)	5.79 (3.2)	8.14 (3.93)	6.91 (3.21)	6.44 (4.48)	6.44 (4.62)	7.08 (2.97)
	Post: Mean (SD)	8.17 (5.38)*	10.71 (4.19)a*	9.32 (3.06)*	8.14 (2.81)*	9.32 (3.57)*	6.25 (2.67)*	8.15 (4.25)*	9.98 (3.85)*	7.79 (3.21)b*
		Group			Group			Group	Gender	
	*F*	2.81			4.46			3.41	4.56	
	*p*	0.08			0.02#			0.04#	0.04#	
	η^2^	0.17			0.25			0.11	0.07	
Motor skill	Pre: Mean (SD)	0.07 (1.13)	0 (1.28)	0.18 (1.19)	−0.02 (1.06)	0.37 (0.92)	0.42 (1.11)	0.03 (1.07)	0.19 (1.09)	0.3 (1.13)
	Post: Mean (SD)	0.55 (0.64)	0.16 (1.16)	0.67 (0.66)	0.52 (0.59)*	0.83 (0.40)	0.85 (0.35)	0.54 (0.60)*	0.51 (0.90)*	0.76 (0.52)
		Group			Group			Group	Gender	
	*F*	0.93			1.07			0.62	2.04	
	*p*	0.41			0.36			0.54	0.16	
	*η^2^*	0.06			0.07			0.02	0.03	
Motor cognition	Pre: Mean (SD)	−1.36 (0.62)	−0.23 (1.16)	−0.38 (1.47)	−1.26 (0.71)	−0.74 (0.88)	−0.77 (0.77)	−1.31 (0.65)	−0.50 (1.03)	−0.58 (1.16)
	Post: Mean (SD)	−0.19 (1.93)	0.82 (0.52)	0.34 (0.80)	0.12 (0.98)	0.99 (0.43)	0.23 (1.14)	−0.05 (1.52)	0.91 (0.47)a*	0.28(0.96)
		Group			Group			Group	Gender	
	*F*	1.11			2.42			3.10	0.60	
	p	0.35			0.11			0.05#	0.44	
	η^2^	0.08			0.15			0.10	0.01	

a, b, *, and # indicate the results of pairwise comparisons. *p < 0.05 compared with the pre-intervention in the *t*-test. * Showing significant difference between pre and post the intervention. a and b represent the results of the LSD multiple comparison test. a: significantly different from FG at the same time point; b: significantly different from FG at the same time point. #*p* < 0.05 indicates the result of an analysis of variance (ANOVA), showing significant differences between groups after the intervention.

When examining the analysis results, the main effect of group on physical fitness was significant (*F* = 4.247, *p* = 0.019, η^2^ = 0.134), indicating meaningful differences among the experimental groups. On the other hand, significant differences in PF variables were observed based on gender (*F* = 6.039, *p* = 0.017, η^2^ = 0.099). Furthermore, no significant interaction effects were found between teaching intervention methods and gender regarding PF variables (*p* > 0.05). As shown in Table 4.

**Table 4:** Main effects comparing the impact of different instructional methods and gender on physical fitness after controlling for pre-test consistency.

**Source**	**Type III sum of square**	**df**	**MS**	***F***	***p***	**η** ^**2**^
Corrected model	416.906a	6	69.484	7.762	<0.001	0.459
Intercept	371.044	1	371.044	41.451	<0.001	0.43
Pre-physical fitness	302.451	1	302.451	33.788	<0.001	0.381
Group	76.037	2	38.019	4.247	0.019	0.134
Gender	54.053	1	54.053	6.039	0.017	0.099
Group * Gender	49.817	2	24.909	2.783	0.071	0.092
Error	492.325	55	8.951			
Total	5552.205	62				
Adj. total	909.23	61				

df, Degrees of Freedom; MS, Mean Square.

Research indicates that participants undergoing specialized SG interventions demonstrated more significant PF gains following instructional interventions, though gender differences persisted. For males, specialized sport skill training may yield greater PF gains, while for females, the method of fitness training intervention appears to be more influential. As shown in Tables 3, 4.

When examining the analysis results, the main effect of participants’ different instructional intervention methods and gender on the impact of motor skill was determined. The main effect of group was also not significant (*p* > 0.05). On the other hand, no significant differences were observed in the MS variable based on gender (*p* > 0.05). Furthermore, no significant differences were found in the MS variable for the interaction between teaching intervention method and gender (*p* > 0.05). As shown in Supplementary Table 3.

Research suggests that participants receiving different instructional interventions exhibited no statistically significant variations in specialized MS development post-intervention. As shown in Table 3 and in Supplementary Table 3.

When examining the analysis results, the main effect of group was determined to have no significant influence on motor cognition due to different instructional intervention methods and gender. The main effect of group was also not significant (*p* > 0.05). On the other hand, no significant differences were observed in the MC variable based on gender (*p* > 0.05). Furthermore, no significant differences were found in the MC variable for the interaction between instructional intervention method and gender (*p* > 0.05). As shown in Supplementary Table 4.

The SG demonstrated the greatest improvement and exhibited significant differences compared to the FG. Research indicates that participants engaged in specialized sports skill learning interventions may exhibit superior improvements in MC compared to other groups following instructional interventions.

### 3.2 Executive function results

First, paired *t*-tests analyzed differences in EF measures across experimental groups before and after intervention. Subsequently, covariance analysis with pre-test scores as covariates examined whether significant differences persisted between groups after controlling for pre-test levels, determining how different PE practices influenced EF measures in junior high students. Subsequently, a covariance analysis was conducted for different genders to examine whether the effects of different intervention methods on students’ EF varied by gender. Finally, a two-way ANOVA was performed to test the interaction effect of intervention method × gender on students’ EF. The results of these analyses, along with relevant tables and charts, are presented below.

When examining the analysis results, determine the main effects of participants’ Stroop ACC, Stroop RT, 2-back ACC and 2-back RT based on different instructional interventions and gender. It was observed that no significant differences existed in Stroop ACC and RT, 2-back ACC and RT variables across instructional intervention, gender, or their interaction (*p* > 0.05). See Supplementary Tables 5–8.

Research indicates that Stroop RT and 2-back RT improved following instructional intervention, yet no significant differences existed between groups in Stroop ACC or RT, 2-back ACC or RT (*p* > 0.05). No significant differences were observed between males and females either (*p* > 0.05). These findings suggest that improvements in IC and WM following instructional intervention may not yield statistically significant differences among participants.

When examining the analysis results, main effects of teaching intervention method and gender on MOS ACC and RT were determined. No significant differences were observed for the MOS ACC variable across teaching intervention method, gender, or their interaction (*p* > 0.05). MOS RT improved following the instructional intervention, with significant differences between groups (*F* = 3.990, *p* = 0.024, η^2^ = 0.127) (Table 5). However, no significant effects were found for gender, instructional method, or the interaction between gender and instructional method (*p* > 0.05). See Table 5 and Supplementary Table 9.

**Table 5:** Main effects comparing the impact of different instructional methods and gender on MOS RT after controlling for pre-test consistency.

**Source**	**Type III sum of square**	**df**	**MS**	***F***	***p***	**η** ^**2**^
Corrected model	3436545.851a	6	572757.642	6.914	<0.001	0.43
Intercept	2790471.619	1	2790471.619	33.685	<0.001	0.38
Pre-MOS RT	1409201.623	1	1409201.623	17.011	<0.001	0.236
Group	661099.596	2	330549.798	3.990	0.024	0.127
Gender	240584.614	1	240584.614	2.904	0.094	0.050
Group * Gender	163607.454	2	81803.727	0.987	0.379	0.035
Error	4556174.960	55	82839.545			
Total	96095697.76	62				
Adj. Total	7992720.811	61				

df, Degrees of Freedom; MS, Mean Square.

However, MOS RT showed significant intergroup differences after the instructional intervention (*F* = 3.58, *p* = 0.03, η^2^ = 0.11). The IG exhibited the most pronounced improvement and demonstrated significant differences compared to the other two groups (*p* < 0.05). Among males, no significant differences existed between groups (*p* > 0.05). Among females, the IG showed significant differences compared to the other two groups (*p* < 0.05). This finding indicates that after the instructional intervention, participants in the interdisciplinary learning intervention showed a more significant improvement in MOS RT. For females, interdisciplinary learning may be more effective in enhancing CF. As shown in Table 6.

**Table 6:** Mixed-effects analysis comparing effects of instructional method and gender on executive function (Stroop ACC, RT; 2-back ACC, RT; MOS ACC, RT) after controlling for pretest consistency.

**Group**	**Variable**	**Male** **(*n* = 31)**			**Female** **(*n* = 31)**			**Total** **(*n* = 62)**		
		**FG** **(*n* = 11)**	**SG** **(*n* = 10)**	**IG** **(*n* = 10)**	**FG** **(*n* = 10)**	**SG** **(*n* = 11)**	**IG** **(*n* = 10)**	**FG** **(*n* = 21)**	**SG** **(*n* = 21)**	**IG** **(*n* = 20)**
Stroop ACC	Pre: Mean (SD)	0.98 (0.03)	0.94 (0.05)	0.97 (0.03)	0.96 (0.03)	0.96 (0.06)	0.99 (0.02)	0.97 (0.03)	0.95 (0.05)	0.98 (0.03)
	Post: Mean (SD)	0.96 (0.03)	0.96 (0.04)	0.98 (0.02)*	0.91 (0.24)	0.91 (0.23)	0.97 (0.03)	0.93 (0.16)	0.93 (0.17)	0.97 (0.03)
		Group			Group			Group	Gender	
	*F*	0.76			0.30			0.45	1.31	
	*p*	0.48			0.74			0.64	0.26	
	η2	0.05			0.02			0.02	0.02	
Stroop RT	Pre: Mean (SD)	906.13 (263.09)	725.2 (122.63)	828.83 (385.76)	991.79 (183.44)	820.67 (202.46)	825.19 (190.64)	946.92 (227.31)	775.21 (172.19)	827.01 (296.16)
	Post: Mean (SD)	760.28 (166.24)*	662.98 (68.64)*	795.28 (352.76)*	820.47 (162.82)	776.31 (154.1)*	716.24 (133.63)*	788.94 (163.39)*	722.35 (131.75)*	755.76 (262.77)*
		Group			Group			Group	Gender	
	*F*	1.46			0.56			0.49	0.00	
	*p*	0.25			0.58			0.61	0.99	
	η2	0.10			0.04			0.02	0.00	
2-back ACC	Pre: Mean (SD)	0.85 (0.06)	0.85 (0.05)	0.87 (0.06)	0.84 (0.05)	0.85 (0.05)	0.84 (0.07)	0.85 (0.06)	0.85 (0.05)	0.86 (0.07)
	Post: Mean (SD)	0.91 (0.04)	0.89 (0.08)	0.9 (0.04)*	0.89 (0.05)	0.83 (0.13)	0.88 (0.07)	0.90 (0.05)*	0.86 (0.11)	0.89 (0.06)
		Group			Group			Group	Gender	
	*F*	0.42			1.12			1.54	2.15	
	*p*	0.67			0.34			0.22	0.15	
	η2	0.03			0.08			0.05	0.04	
2-back RT	Pre: Mean (SD)	853.93 (171.38)	910.95 (137.3)	892.64 (158.41)	914.90 (127.51)	979.72 (172.93)	956.63 (122.17)	882.96 (151.58)	946.97 (157.08)	924.63 (141.54)
	Post: Mean (SD)	841.18 (219.74)*	828.76 (134.36)*	896.72 (148.98)	856.80 (161.36)	934.14 (107.6)	877.7 (197.76)	848.62 (189.54)	883.96 (129.70)*	887.21 (170.69)
		Group			Group			Group	Gender	
	*F*	0.78			0.48			0.09	0.03	
	*p*	0.47			0.62			0.91	0.87	
	η2	0.05			0.04			0.00	0.00	
MOS ACC	Pre: Mean (SD)	0.91 (0.11)	0.88 (0.10)	0.91 (0.10)	0.91 (0.06)	0.90 (0.11)	0.83 (0.16)	0.91 (0.09)	0.89 (0.11)	0.87 (0.13)
	Post: Mean (SD)	0.93 (0.08)*	0.91 (0.11)	0.91 (0.09)	0.91 (0.07)	0.86 (0.16)	0.88 (0.14)	0.92 (0.07)	0.88 (0.14)	0.89 (0.11)
		Group			Group			Group	Gender	
	F	0.13			0.42			0.53	1.09	
	p	0.88			0.66			0.59	0.30	
	η2	0.01			0.03			0.02	0.02	
MOS RT	Pre: Mean (SD)	1351.36 (400.49)	1359.74 (349.96)	1300.34 (563.9)	1645.04 (546.74)	1527.44 (509.84)	1225.19 (351.53)	1491.21 (487.13)	1447.58 (438.69)	1262.76 (458.96)
	Post: Mean (SD)	1119.23 (378.89)*	1187.49 (265.38)*	1008.33 (196.9)*	1468.9 (322.57)	1381.51 (496.09)	975.27 (128.92)*	1285.74(388.1)*	1289.12 (405.71)*	991.80 (162.87)ab*
		Group			Group			Group	Gender	
	F	1.56			3.53			3.58	2.40	
	p	0.23			0.04#			0.03#	0.13	
	η2	0.10			0.21			0.11	0.04	

a, b, *, and # indicate the results of pairwise comparisons. *p < 0.05 compared with the pre-intervention in the *t*-test. * Showing significant difference between pre and post the intervention. a and b represent the results of the LSD multiple comparison test. a: significantly different from FG at the same time point; b: significantly different from FG at the same time point. #*p* < 0.05 indicates the result of an analysis of variance (ANOVA), showing significant differences between groups after the intervention.

### 3.3 Correlation analysis results

Analysis of the correlation results revealed that 2-back RT correlated with sub-indicators of MA, showing a significant negative correlation with PF (*r* = −0.290, *p* = 0.022) and a significant positive correlation with MC (*r* = 0.299, *p* = 0.018). To further examine the relationship between MA and EFs following different interventions, the following findings emerged: (1) After specialized sports skills training intervention, 2-back ACC showed a significant positive correlation with MA (*r* = 0.449, *p* = 0.041), while 2-back RT demonstrated a significant positive correlation with MA (*r* = 0.492, *p* = 0.023), and MOS ACC showed a significant negative correlation with MS (*r* = −0.485, *p* = 0.026); (2) Following interdisciplinary learning intervention, Stroop RT showed a significant negative correlation with MC (*r* = −0.534, *p* = 0.015), and MOS ACC showed a significant negative correlation with MC (*r* = −0.445, *p* = 0.049). Additionally, for female participants, Stroop ACC positively correlated with MS (*r* = 0.361, *p* = 0.046), and 2-back RT positively correlated with MS (*r* = 0.375, *p* = 0.038).

## 4 Discussion

The objective was to explore differences in MA (PF, MS, and MC) among students participating in different types of PE courses, considering various intervention methods and demographic variables such as gender.

### 4.1 Motor ability discussion

First, the study found that students’ motor ability improved across all types of physical education interventions, with significant differences observed (*p* < 0.05). Students who participated in specialized sports skill training demonstrated the most pronounced overall improvement in MA. For female students, these intervention-related differences in MA were more pronounced (*p* < 0.05), but this does not indicate a statistically significant gender difference. This is because the study found no significant differences based on gender, nor any significant interaction between the type of teaching intervention and gender (see Table 3). A few studies have reported similar results. This result may stem from specialized motor skills instruction being more targeted in enhancing students’ MA compared to the other two approaches. Engaging in physical activities through various environment-specific movement experiences can promote MA development. Overall, a positive relationship exists between physical activity interventions and MA (39). Several studies have demonstrated findings similar to this research. The motor competence assessed in this study encompasses a broad range of components, including PF, MS, and MC. Multiple investigations have confirmed that after learning sport-specific skills, students exhibit improvements in motor competence-related indicators. Existing literature suggests that structured skill instruction effectively enhanced students’ self-perceived motor competence and cardiorespiratory endurance. Incorporating competitive formats increased interest in PE (40). Specialized skill training methods were more effective in boosting students’ engagement in PE and enhancing their achievement motivation. Researchers found that students’ MA levels are closely related to their PE learning motivation. The fulfillment of psychological needs appears to exert direct or indirect effects on children’s PE learning motivation and skill development. The study revealed more pronounced intergroup differences among female students following interventions with different instructional content (41). The study found that girls demonstrated increased intentions to engage in more physical exercise after the intervention. This may explain the greater changes in girls’ motor skills (42).

From the perspective of the Skill-Theme, PE instruction organized around specific movement themes and sport skills enables students to repeatedly practice fundamental movement patterns within meaningful game contexts. This approach allows learners to integrate motor skills with tactical understanding and decision-making, thereby facilitating more effective development of MA. A study proves that as students practice forming different shapes in gymnastics, they develop a deeper understanding of how their bodies move and interact with space, which is crucial for sequence, jumping over obstacles, and buoyant and yielding landings (43). This result may be explained by Neuromotor Synergy Theory. In complex motor tasks (such as sport skills or competitive scenarios), multiple joints and muscles must coordinate simultaneously. This synergistic mechanism can facilitate more efficient motor control and physiological adaptation, (44) which may contribute to improvements in students’ MA.

In terms of physical fitness indicators, all groups demonstrated improved fitness levels post-intervention, with significant differences observed (*p* < 0.05). The specialized sports skills training intervention yielded more pronounced PF changes among students, with significant gender differences (*p* < 0.05). Female participants exhibited more pronounced PF changes following the intervention (*p* < 0.05). However, the study found no significant interaction between group and gender (Table 3). This outcome may stem from the fact that while PF training targets students’ physical capabilities more directly, specialized sports skill training better engages students in complex athletic scenarios like competitions, thereby further enhancing PF. Neuromotor Synergy Theory (44) may explain why complex athletic scenarios yield superior fitness gains compared to traditional methods. Multiple studies provide evidence for PF improvements. Researchers found that after an 11-week soccer intervention for children aged 9–12, students demonstrated significant improvements in PF indicators such as body composition compared to the control group, specifically in body fat percentage and lean body mass (*p* < 0.01) (7). A study demonstrated that properly guided participation in physical activities during PE classes yields superior effects on cardiorespiratory endurance compared to routine physical activity (11). Others found through a study of 195 elementary school children in North Texas that children’s expectancy beliefs were significantly correlated with motor skills (*R*^2^ = 11%) and cardiorespiratory fitness (*R*^2^ = 16%) (45). A study found that moderate-to-high intensity physical activities in PE classes were more effective for improving muscle strength (12). Another found that engaging physical activities produced more significant improvements in students’ speed, cardiorespiratory fitness, strength, flexibility, agility, and coordination, leading to overall positive changes in PF (15). Similar results emerged from a Danish study by researchers found that after implementing the 11 for Health in Denmark program—which included health education, football drills, and small-sided games—the experimental group showed increased physical activity (+5.9 percentage points, 95% CI 4.1 to 7.7%, ES: 0.36) compared to the control group. This rise in physical activity may be a key factor in the positive changes observed in PF (46). In another study found that girls were more likely than boys to increase moderate-to-vigorous physical activity (MVPA) levels at the 12-month follow-up (47). Researchers also found that boys showed reduced participation in unstructured physical activities, while girls’ participation remained stable (48). This may explain why girls’ groups exhibited more pronounced changes following instructional interventions.

Motor skill improved following instructional intervention, with no significant differences observed across groups, between males and females, or in the interaction between intervention method and gender (Table 3). A study found that MS can only reach a proficient level through complex competitive matches and repeated practical application (49). In our study, we observed that many students demonstrated relatively high MS proficiency in the pre-test, potentially influenced by age and prior learning foundations, resulting in limited differences between pre- and post-tests. Additionally, the selected MS assessment included match scoring, where multiple students in team sports like soccer might achieve identical scores—a factor contributing to the lack of significant differences. Furthermore, the intervention period in this study lasted only 12 weeks. The insufficient duration of the intervention and the lack of follow-up data also contributed to the lack of significant differences in MS.

Motor cognition improved significantly after the instructional intervention, with significant differences observed across groups (*p* < 0.05). The specialized sports skill training intervention brought positive changes to students. However, no significant differences were found in gender, intervention method, or the interaction between gender and intervention (Table 3). Previous studies indicate that only increased participation in competitions and practice can enhance students’ perception of sports. Learning sport-specific skills provided students with more opportunities to participate in competitions, which may explain the improvement in MC following the intervention (50).

### 4.2 Executive function discussion

Inhibitory control improved following instructional intervention, with no significant differences across groups, between males and females, or in the interaction between intervention method and gender (Table 7). Multiple studies align with the structure of this research found no significant differences between the intervention and control groups in the Stroop task’s time effects or the interaction between time and intervention method in an Enriched Sports Activity Program (ESA Program) intervention involving 357 children across four countries (51). Researchers found no significant changes in IC among children with intellectual disabilities following a 6-week intensive PE program (52). A study observed no significant differences in IC among preschool children after a structured physical training intervention targeting basic motor skills (53). A recent systematic review found that short-duration, cognitively engaging exercises—such as rhythm-based activities or task-switching drills—are often associated with immediate improvements in IC. Furthermore, long-term interventions yield positive effects on both IC and WM (54). Intervention duration may also be one reason for the lack of significant differences between groups in the teaching intervention.

**Table 7:** Correlation analysis results exploring the relationship between motor ability and executive function.

**Participants**	**Variables**		**Fitness**	**Motor cognition**	**Motor skill**	**Motor ability**
Total	2-back RT	*r*	−0.290*	0.299*		
		*p*	0.022	0.018		
FG	2-back ACC	*r*				0.449*
		*p*				0.041
	2-back RT	*r*			0.492*	
		*p*			0.023	
	MOS ACC	*r*			−0.485*	
		*p*			0.026	
IG	Stroop RT	*r*		−0.534*		
		*p*		0.015		
	MOS ACC	*r*		−0.445*		
		*p*		0.049		
Female	Stroop ACC	*r*			0.361*	
		*p*			0.046	
	2-back RT	*r*			0.375*	
		*p*			0.038	

* Indicates results with significant correlation.

Working memory improved across all groups following the instructional intervention. Significant improvements were observed in both the FG and SG. No significant differences were found between groups, between males and females, or in the interaction between intervention type and gender. A study investigated the effects of different types of cognitively challenging physical interactions on students’ EF. They found that all types had positive effects on the EF of students in the experimental group, but no significant differences were observed between groups (55).

There is no significant group difference in inhibitory control and working memory, which may be due to insufficient cognitive engagement or the lack of distinct differentiation in cognitive load settings between the groups, leading to statistically insignificant results. Effectiveness of the cognitive challenging physical activity games for triggering students’ EFs in PE. A study found that students who played the cognitively challenging physical activity games reported higher scores on novelty compared to students in the soccer or track and field groups (72).

Cognitive flexibility improved following the instructional intervention. Although no between-group differences were observed for MOS ACC, significant between-group differences emerged for MOS RT post-intervention. The IG demonstrated the most pronounced improvement, exhibiting significant differences compared to the other two groups (*p* < 0.05). Among males, no significant differences existed between groups (*p* > 0.05). Among females in the IG, significant differences were observed compared to the other two groups (*p* < 0.05). This finding indicates that participants engaged in interdisciplinary learning interventions demonstrated more pronounced improvements in MOS RT following instructional interventions. For females, interdisciplinary learning may yield more significant gains in CF.

The integration of different disciplines likely required participants to switch between different cognitive tasks more frequently, thus engaging more dynamic and flexible thinking processes. In research on executive functions, cognitive flexibility does not remain constant. It is influenced by the prefrontal cortex, as well as factors such as the availability of cognitive resources, emotional state, and motivation levels, and can fluctuate (56). Neuroimaging studies on task switching reveal that numerous brain regions are activated additionally when subjects prepare to switch tasks and execute the changed task (57). This finding is consistent with previous research. They demonstrated that more varied physical exercise yields superior cognitive flexibility outcomes (58). A study found that PA with higher cognitive load yielded better effects, while other studies reported no differences (59). These findings align with our results, indicating that interdisciplinary learning effectively engages richer cognitive activities in students’ learning processes. Researchers discovered that increased reflective thinking effectively influences cognitive flexibility. In PE classes, students’ reflective behaviors and other cognitive processes are further activated, leading to enhanced CF (60). A study observed that after a 10-week group PE intervention involving moderate-to-high intensity training and complex scenarios with mini-games, students demonstrated significantly greater improvements in CF compared to the control group (61). This finding aligns with our study, where students engaged in more complex movement contexts during interdisciplinary learning, leading to more pronounced CF gains.

### 4.3 Correlation discussion

Another one objective of this study was to explore correlations between students’ motor ability and executive functions. Our findings indicate that post-intervention, students’ PF, MC, and WM levels showed positive correlations. Female students exhibited a positive correlation between MS and IC, but a negative correlation with WM levels. Following the physical fitness learning intervention, students’ MA correlated positively with WM levels, while MS correlated negatively with both WM and CF levels. Following interdisciplinary learning, students’ MC positively correlated with WM but negatively correlated with ACC in CF, while RT in CF decreased. These findings partially align with previous research.

A study found statistically significant positive correlations between physical health and EF (*β* = 0.28, 95% CI [0.04, 0.52]) and between EF and academic achievement (*β* = 0.46, 95% CI [0.28, 0.65]) (62). Researchers found a positive correlation between EF and MA, a relationship constrained and influenced by individual gender and environment (63). This may relate to differing levels of participation in physical activities among students of different genders. While previous research has not extensively examined the relationship between MA and student EF, numerous studies have explored various sub-indicators and dimensions of MA. A study found that students exhibiting higher levels of PF also demonstrated superior development in EF such as WM and IC (64). Researchers found in a study of 896 schizophrenia outpatients that higher BMI was significantly associated with lower cognitive composite *Z*-scores (*p* < 0.05) (65). Another one found in a study of 263 children aged 7–12 that PF was correlated with EF (*r* = 0.430, *R*^2^ = 0.190) in a structural equation model linking PF to EF and academic performance (66). A cross-sectional study also provided supporting evidence. A study found significant negative correlations between cardiorespiratory endurance and IC (beta = −0.14, *p* < 0.05), and significant positive correlations between lower-body explosive power and IC (beta = 0.13, *p* < 0.05) (67).

Another potential explanation relates to the specificity of the assessment tasks used to measure both motor skills and executive functions. There may be deviations from previous studies, which may be caused by acute stress. When students face tasks, they may experience greater pressure, potentially leading to a decrease in their cognitive abilities. Acute stress may lead to a decline in higher-order cognitive functions (68). Another reason for these results may be related to the inefficient reallocation of attention. When students engage in motor skill tasks that require higher levels of coordination and motor control, a substantial amount of cognitive resources may be allocated to movement planning and execution, thereby temporarily reducing the resources available for executive function processes such as WM updating or CF. A study indicate that individual creativity, as measured by the divergent thinking test, is related to the inefficient reallocation of attention, congruent with the idea that diffuse attention is associated with individual creativity (69).

Multiple studies further elucidate the relationship between EF and PF. Complex PE activities offer potential for enhancing children’s cognitive development. A study proposed that thinking while moving may yield greater effects for children’s cognitive training. Additionally, physical movement compels the brain to operate more efficiently, and in certain contexts, motor activities may serve as necessary compensatory mechanisms to promote cognitive function (70). They found that diverse and engaging physical activities uniquely promote both motor and cognitive development. Their study revealed that rich, interesting PE programs benefit IC of cognitive EFs but do not significantly enhance WM updating or attention. This indicates distinct associations between specific aspects of MA and particular executive processes (27). Furthermore, research revealed that soccer players’ performance during matches and their judgment of tactics and techniques positively correlate with EF, as well as with skill level (71).

Overall, these findings suggest that while motor ability and executive function are generally positively related, the relationship may vary depending on the specific motor components, cognitive processes, and contextual demands involved.

### 4.4 Strengths and limitations of the study

A key strength of this study lies in its examination of changes in students’ motor ability, executive function, and their sub-indicators following participation in different types of physical education classes. It innovatively employed *Z*-scores to measure MA. Another methodological strength is the use of *Z*-scores to assess MA. By standardizing different physical indicators onto a common scale, the use of *Z*-scores allows for more robust comparisons across diverse physical metrics, reducing the influence of differences in measurement units and improving the interpretability of composite MA indicators. Furthermore, the research uniquely explored the relationship between MA and EF, investigating how different instructional interventions influence the correlation between these two domains in students.

The primary limitation of this study lies in its 12-week intervention period without follow-up or long-term impact assessment. The sample was restricted to junior high school students, limiting the exploration of age diversity; expanding the demographic and sample diversity warrants consideration in future research. The study did not conduct further analysis of other sub-indicators within MA, such as PF, MC, and MS. Future research should incorporate more indicators for detailed comparative analysis. Furthermore, while the correlation between MA and EF was examined, suggesting a relationship between the two, the study did not delve into influencing factors. This finding should be interpreted with caution but also provides direction for future research, such as investigating these influencing factors.

A limitation of this study is the lack of data on physical activity levels. In this study, the Polar device was used to assess exercise intensity only during the initial class. However, objective monitoring methods such as accelerometers or continuous heart-rate monitoring were not employed throughout the entire intervention to evaluate exercise intensity, which may represent an important confounding factor influencing the development of executive functions. Future research should incorporate objective physical activity monitoring devices, such as accelerometers, to control for its influence on both MA and EF. This study did not further investigate the mechanisms underlying the correlation between them.

Some issues arising from the study design and factors not considered during the experimental process are indeed limitations of this research. This study did not calculate Cohen’s kappa, which is a limitation. Students who were ill were given only the baseline score, and this may indeed have introduced noise into the data. The possibility of observer bias and measurement bias during the grouping process by testers is also one of the limitations of this study. In response to the limitations identified in the above studies, we will adopt a more rigorous and standardized research design in future investigations.

### 4.5 Future research directions

To address these limitations, future studies should conduct in-depth longitudinal research, extend intervention durations, or implement follow-ups to establish causal relationships between MA and EF. Future studies may also consider broadening participant diversity by recruiting students from different regions and age groups. Additionally, expanding measurement to include environmental factors, parental education, extracurricular activities, and sociocultural aspects will provide a more comprehensive explanation for potential causes of MA and EF changes. Furthermore, incorporating objective physical activity data is essential to control for confounding effects of physical activity levels on MA and EF. Future research should also focus on investigating the mechanisms underlying the relationship between them, such as brain mechanisms, mediating effects, or moderating effects, to provide additional evidence.

## 5 Conclusion

This study examined the effects of different physical education teaching interventions on students’ motor ability and executive functions, as well as the relationship between these two domains. All three teaching interventions enhanced students’ MA and EF, with the most pronounced changes observed among students participating in specialized sports skill learning. Further analysis indicates that specialized sports skill training brought about more significant changes in PF and MC. Among them, interdisciplinary learning produced more pronounced changes in CF. PE courses involving high uncertainty and complex cognitive tasks (such as interdisciplinary thematic learning) more effectively promoted advanced cognitive functions in junior high school students. Another key finding was the significant correlations between MA sub-indicators and EF: students’ PF, MC, and WM exhibited significant positive correlations. The study found that girls’ MS positively correlated with IC but negatively correlated with WM. After physical training, students’ MA positively correlated with WM levels, while MS negatively correlated with WM and CF. Following interdisciplinary learning, students’ MC showed a significant positive correlation with WM, which suggesting that physical development and cognitive functions are closely interconnected.

These findings have important implications for school-based physical education and adolescents’ public health. We have discovered that different types of PE curriculum content play distinct roles in students’ healthy development. School PE should integrate diverse instructional approaches, including physical conditioning, sports skill learning, and interdisciplinary activities that combine physical movement with cognitive challenges. Such approaches may simultaneously promote motor competence and higher-order cognitive functions in students. It should focus on students applying diverse knowledge and skills to solve problems, integrating comprehensive knowledge and skills into activity design. Emphasizing MA development in physical activities can enhance students’ cognitive functions. Incorporating interactive elements of physical and cognitive exercises into PE curricula strengthens the connection between MA and EF. This includes adding games requiring quick decision-making, increasing segments where multidisciplinary knowledge solves movement challenges, and conducting exercises or competitions that activate cognitive and MS while requiring teamwork. For education policymakers and school administrators, designing PE programs that incorporate structured physical training, skill-based sports learning, and cognitively engaging activities may support both physical health and cognitive development among adolescents.

Furthermore, to validate these findings, future research should extend intervention durations. It should also broaden participant diversity, increase sample variability, and design tailored interventions for distinct demographic characteristics. Such interventions may incorporate adaptive and challenging training activities to enhance students’ MA and EF. Research indicates that multiple factors influence motor skills and cognitive development. To further validate these findings, future studies should incorporate multifactorial analyses encompassing environmental factors, family influences, and extracurricular activities. Additionally, experimental designs establishing causal relationships between MA and EF should be developed to deepen understanding of underlying mechanisms. Further research should also explore how cultural and environmental factors shape these relationships across diverse populations, enabling more targeted policy recommendations.

## Data Availability

The raw data supporting the conclusions of this article will be made available by the authors, without undue reservation.

## References

[1] Stodden DF Goodway JD Langendorfer SJ Rberton MA Rudisill ME Garcia C . A developmental perspective on the role of motor skill competence in physical activity: an emergent relationship. Quest. (2008) 60:291–306. doi: 10.1080/00336297.2008.10483582

[2] U.S. Department of Health and Human Services. Physical Activity Guidelines for Americans. 2nd ed. (2018) Washington, DC: U.S. Department of Health and Human Services. p. 33–9.

[3] Sarrazin P Biddle S Famose JP Cury F Fox K Durand M. Goal orientations and conceptions of the nature of sport ability in children: a social cognitive approach. Br J Soc Psychol. (1996) 35:399–414. doi: 10.1111/j.2044-8309.1996.tb01104.x

[4] Ji L. An interpretation of the physical education and health curriculum standards for compulsory education (2022 edition) in China. Sports Sci. (2022) 42:3–17. doi: 10.16469/j.css.202205001

[5] Shi P Xin F Zhang DY Zha XL Wang WC Jin T . A theoretical exploration of the benefits of physical exercise for executive function in children and adolescents. J Wuhan Institute Physical Educ. (2023) 57:75–83. doi: 10.15930/j.cnki.wtxb.2023.11.009

[6] Li M. H. (2006) Executive function development in children and adolescents across grades and academic achievement levels. Dissertation, Tianjin (China): Tianjin Normal University

[7] Zhu JM Lü JD Long YY Xu Q. Effects of the “FIFA 11 for health” football curriculum intervention on physical fitness in children aged 9–12 years. J Shanghai Univers Sport. (2022) 46:72–81. doi: 10.16099/j.sus.2021.07.19.0003

[8] Wang S. X. (2019) Effects of a 12-week school football training program on physical fitness in 9–10-year-old boys. Master’s thesis, Tianjin (China): Tianjin University of Sport

[9] Luo K. (2016) An experimental study on the effects of a 10-week football intervention on physical fitness in middle school students. Master’s thesis. Yangzhou (China): Yangzhou University

[10] Wang C Yuan Y Ji X. Effects of blended learning in physical education on university students’ exercise attitudes and basketball skills: a cluster randomized controlled trial. BMC Public Health. (2024) 24:3170. doi: 10.1186/s12889-024-20469-x, 39543547 PMC11566481

[11] Calahorro-Cañada F Torres-Luque G López-Fernández I Carnero EA. Is physical education an effective way to increase physical activity in children with lower cardiorespiratory fitness? Scand J Med Sci Sports. (2017) 27:1417–22. doi: 10.1111/sms.12740, 27466085

[12] Lemes VB Gaya ACA Brand C Dias AF Cristi-Montero C Mota J . Associations among psychological satisfaction in physical education, sports practice, and health indicators with physical activity. Int J Pediatr Adolesc Med. (2021) 8:246–52. doi: 10.1016/j.ijpam.2020.11.004, 34401450 PMC8356103

[13] Ji HJ Liang YH Wei MT Wang XL. Grey relational analysis of physical fitness in 11-year-old boys during the sensitive period for speed development. J Beijing Sport Univ. (2006) 29:804–6. doi: 10.19582/j.cnki.11-3785/g8.2006.06.028

[14] Chen F. L. (2018) Effects of combined motor-skill and physical-fitness practice in physical education on children’s and adolescents’ physical and mental health. Dissertation, Shanghai (China): East China Normal University

[15] Melero-Cañas D Morales-Baños V Manzano-Sánchez D Navarro-Ardoy D Valero-Valenzuela A. Effects of an educational hybrid physical education program on physical fitness, body composition and sedentary and physical activity times in adolescents: the Seneb’s enigma. Front Psychol. (2021) 11:629335. doi: 10.3389/fpsyg.2020.629335, 33510699 PMC7835141

[16] Siersbaek GM Have M Wedderkopp N. The effect of leisure time sport on executive functions in Danish 1st grade children. Children. (2022) 9:1458. doi: 10.3390/children9101458, 36291397 PMC9601112

[17] Jiang CH Yan SF Wang SH. Physical activity promotes brain executive function: behavioral, EEG and MRI evidence. Chin J Sports Med. (2012) 31:636–41. doi: 10.16038/j.1000-6710.2012.07.014

[18] Zhou Z Dong S Yin J Fu Q Ren H Yin Z. Improving physical fitness and cognitive functions in middle school students: study protocol for the Chinese childhood health, activity and motor performance study (Chinese CHAMPS). Int J Environ Res Public Health. (2018) 15:976. doi: 10.3390/ijerph15050976, 29757933 PMC5982015

[19] Yin HC Chen AG Ma Z Li XN Liu M. A follow-up study on the effects of two exercise intervention programs on executive function in primary school students. Sports Sci. (2014) 34:24–8. doi: 10.16469/j.css.2014.03.001

[20] Yang L Corpeleijn E Hartman E. Daily physical activity, sports participation, and executive function in children. JAMA Netw Open. (2024) 7:e2449879. doi: 10.1001/jamanetworkopen.2024.49879, 39688868 PMC11653117

[21] Ishihara T Sugasawa S Matsuda Y Mizuno M. The beneficial effects of game-based exercise using age-appropriate tennis lessons on the executive functions of 6–12-year-old children. Neurosci Lett. (2017) 642:97–101. doi: 10.1016/j.neulet.2017.01.057, 28159634

[22] Ishihara T Kuroda Y Mizuno M. Competitive achievement may be predicted by executive functions in junior tennis players: an 18-month follow-up study. J Sports Sci. (2019) 37:755–61. doi: 10.1080/02640414.2018.1524738 30332916

[23] Chen AG Zhao L Li HY Yan J Yin HC. Effects of short-duration basketball dribbling training at different intensities on executive function in primary school students. J Tianjin Univ Sport. (2014) 29:352–5. doi: 10.13297/j.cnki.issn1005-0000.2014.04.015.

[24] Ren Y Chu J Zhang Z Luo B. Research on the effect of different aerobic activity on physical fitness and executive function in primary school students. Sci Rep. (2024) 14:7956. doi: 10.1038/s41598-024-58009-7, 38575618 PMC10995128

[25] Vorraber Lawson G Ugrinowitsch C Costa R Lamas L. Effects of different types of chronic physical activities and sports on executive functions among children and adolescents: a systematic review and meta-analysis. J Sports Sci. (2025) 43:565–79. doi: 10.1080/02640414.2025.2468587, 39967007

[26] Mavilidi M Lubans D Eather N Morgan P Riley N. Preliminary efficacy and feasibility of “thinking while moving in English”: a program with physical activity integrated into primary school English lessons. Children. (2018) 5:109. doi: 10.3390/children5080109, 30103471 PMC6111322

[27] Pesce C Masci I Marchetti R Vazou S Sääkslahti A Tomporowski PD. Deliberate play and preparation jointly benefit motor and cognitive development: mediated and moderated effects. Front Psychol. (2016) 7:349. doi: 10.3389/fpsyg.2016.00349, 27014155 PMC4786558

[28] Egger F Benzing V Conzelmann A Schmidt M. Boost your brain, while having a break! The effects of long-term cognitively engaging physical activitybreaks on children's executive functions and academic achievement. PLoS One. (2019) 14:e0212482. doi: 10.1371/journal.pone.0212482, 30840640 PMC6402646

[29] Contreras-Osorio F Campos-Jara C Martínez-Salazar C Chirosa-Ríos L Martínez-García D. Effects of sport-based interventions on children’s executive function: a systematic review and meta-analysis. Brain Sci. (2021) 11:755. doi: 10.3390/brainsci11060755, 34200362 PMC8226694

[30] Brioa Saez G Rico-González M Monge Gómez N. The effect of physical-activity-based programs on school children’s cognitive competence-related variables: a systematic review of randomized controlled trials. Sports. (2025) 13:261. doi: 10.3390/sports13080261, 40863770 PMC12390218

[31] Álvarez-Bueno C Pesce C Cavero-Redondo I Sánchez-López M Martínez-Hortelano JA Martínez-Vizcaíno V. The effect of physical activity interventions on children’s cognition and metacognition: a systematic review and meta-analysis. J Am Acad Child Adolesc Psychiatry. (2017) 56:729–38. doi: 10.1016/j.jaac.2017.06.012, 28838577

[32] Zhou CL Jin XH. How physical exercise enhances learning benefits from a brain science perspective: theory and practice. J Shanghai Univ Sport. (2021) 45:20–8. doi: 10.16099/j.sus.2021.01.003

[33] Fernandes V Silva A Carvalho A Ribeiro S Deslandes A. Physical fitness, executive functions, and academic performance in children and youth: a cross-sectional study. Behav Sci. (2024) 14:1022. doi: 10.3390/bs14111022, 39594323 PMC11591446

[34] Cao L-Z He H Miao X Chi L. The contributions of executive functions to decision-making in sport. Int J Sport Exerc Psychol. (2024) 23:1066–85. doi: 10.1080/1612197X.2024.2371483

[35] Fernández-Sánchez A Ruiz-Hermosa A Redondo-Tébar A Díez-Fernández A Martínez-Vizcaíno V Visier-Alfonso ME . Relation between motor competence and academic achievement: the mediating role of fitness and cognition in boys and girls. PLoS One. (2025) 20:e0314948. doi: 10.1371/journal.pone.0314948, 41202042 PMC12594380

[36] Ji L Yin XJ Wu HP Yang XF Liu Y. Exploring standards for evaluating physical fitness in Chinese children and adolescents under the integration of sports and education context. Sports Science. (2021) 41:42–54. doi: 10.16469/j.css.202103006

[37] Dai S. T.. (2021). Experimental study on the effects of the Chinese Healthy physical education curriculum model on core competencies in physical education and health among primary and secondary school students. Dissertation, Shanghai (China): East China Normal University

[38] Yang X Yin X Huang W Duan Z Wu H Li Y . Distribution of cardiorespiratory fitness in children and adolescents at different latitudes. Am J Hum Biol. (2023) 35:e23908. doi: 10.1002/ajhb.23908, 37212366

[39] Gu X Fu Y Chen W Tamplain PM Zhang T Wang J. A causal pathway of physical activity to motor competence in childhood: evidence from a school-based intervention. J Sports Sci. (2021) 39:460–8. doi: 10.1080/02640414.2020.1826666, 33019892

[40] Cai RJ Bao HY Ji L. Associations between structured skill teaching and adolescents’ sport self-concept and interest in physical education learning. J Chengdu Sport Univers. (2022) 48:104–10. doi: 10.15942/j.jcsu.2022.05.017

[41] de Bruijn AGM Mombarg R Timmermans AC. The importance of satisfying children’s basic psychological needs in primary school physical education for PE-motivation, and its relations with fundamental motor and PE-related skills. Phys Educ Sport Pedagog. (2022) 27:422–39. doi: 10.1080/17408989.2021.1906217

[42] Ferrer M Camerino O Castañer M. Hybridizing pedagogical models to foster personal responsibility in improving physical fitness in adolescents. Apunts Educ Fis Deporte. (2025) 162:19–30. doi: 10.5672/apunts.2014-0983.es.(2025/4).162.03

[43] McMurray A Ison S Wahl-Alexander Z. A skill theme approach to teaching fundamental motor skills through gymnastics-based activities. J Phys Educ Recreat Dance. (2025) 96:6–13. doi: 10.1080/07303084.2025.2463900

[44] Tessari F West AM Hogan N. Explaining human motor coordination via the synergy expansion hypothesis. Proc Natl Acad Sci USA. (2025) 122:e2501705122. doi: 10.1073/pnas.2501705122, 40146855 PMC12002196

[45] Zhang X Gu X Chen S Keller MJ Lee J. The roles of sex and minority status in children’s motivation and psychomotor learning. Percept Mot Skills. (2021) 128:2849–66. doi: 10.1177/00315125211046446, 34514897

[46] Larsen MN Elbe A-M Madsen M Madsen EE Ørntoft C Ryom K . An 11-week school-based health education through football programme improves health knowledge related to hygiene, nutrition, physical activity and well-being—and it is fun! Br J Sports Med. (2021) 55:906–11. doi: 10.1136/bjsports-2020-103097 33509911

[47] Huang W Shi X Wang Y Li X Gao P Lu J . Determinants of students’ physical activity: a 12-month follow-up study in Ningxia province. BMC Public Health. (2021) 21:512. doi: 10.1186/s12889-021-10525-1, 33726744 PMC7968158

[48] Kemp BJ Cliff DP Chong KH Parrish A-M. Longitudinal changes in domains of physical activity during childhood and adolescence: a systematic review. J Sci Med Sport. (2019) 22:695–701. doi: 10.1016/j.jsams.2018.12.012, 30630743

[49] Wang XZ. Content structure and features of the physical education and health curriculum standards for compulsory education (2022 edition). J Capital Univers Physical Educ Sports. (2022) 34:241–252, 274. doi: 10.14036/j.cnki.cn11-4513.2022.03.003

[50] Lee AM Fredenburg K Belcher D Cleveland N. Gender differences in children’s conceptions of competence and motivation in physical education. Sport Educ Soc. (1999) 4:161–74. doi: 10.1080/1357332990040204

[51] Gentile A Boca S Şahin FN Güler Ö Pajaujiene S Indriuniene V . The effect of an enriched sport program on children’s executive functions: the ESA program. Front Psychol. (2020) 11:657. doi: 10.3389/fpsyg.2020.00657, 32411039 PMC7198739

[52] Mero Piedra AL Pesthy O Marton K. Effects of a physical education intervention on attention and inhibitory control in Ecuadorian children with intellectual disabilities. J Intellect Disabil. (2024) 28:261–74. doi: 10.1177/17446295231189018, 37458606

[53] Şendil AM Canlı U Sheeha BB Alkhamees NH Batrakoulis A Al-Mhanna SB. The effects of structured coordinative exercise protocol on physical fitness, motor competence and inhibitory control in preschool children. Sci Rep. (2024) 14:28462. doi: 10.1038/s41598-024-79811-3, 39558052 PMC11574278

[54] González-del-Castillo J Barbero-Alcocer I. Effects of school-based physical activity programs on executive function development in children: a systematic review. Front Psychol. (2025) 16:1658101. doi: 10.3389/fpsyg.2025.1658101, 40969463 PMC12440757

[55] Kolovelonis A Goudas M. Exploring the effects of three different types of cognitively challenging physical activity games on students’ executive functions and situational interest in physical education. Cogent Educ. (2022) 9:2148448. doi: 10.1080/2331186X.2022.2148448 PMC10217652 37232698

[56] Diamond A. Executive functions. Annu Rev Psychol. (2013) 64:135–68. doi: 10.1146/annurev-psych-113011-143750, 23020641 PMC4084861

[57] Monsell S. Task switching. Trends Cogn Sci. (2003) 7:134–40. doi: 10.1016/S1364-6613(03)00028-7, 12639695

[58] Tocci N Scibinetti P Mazzoli E Mavilidi MF Masci I Schmidt M . Giving ideas some legs or legs some ideas? Children’s motor creativity is enhanced by physical activity enrichment: direct and mediated paths. Front Psychol. (2022) 13:806065. doi: 10.3389/fpsyg.2022.806065, 35360626 PMC8960453

[59] Bossio M Justel N. Working memory, inhibition and cognitive flexibility: modulation through physically activating interventions. Interdisciplinaria. (2023) 40:18–9. doi: 10.16888/interd.2023.40.3.18

[60] Orakcı Ş. Exploring the relationships between cognitive flexibility, learner autonomy and reflective thinking. Think Skills Creat. (2021) 41:100838. doi: 10.1016/j.tsc.2021.100838

[61] Ángel Latorre-Román P Berrios-Aguayo B Aragón-Vela J Pantoja-Vallejo A. Effects of a 10-week active recess program in school setting on physical fitness, school aptitudes, creativity and cognitive flexibility in elementary school children: a randomised-controlled trial. J Sports Sci. (2021) 39:1277–86. doi: 10.1080/02640414.2020.1864985, 33407022

[62] Muntaner-Mas A Mazzoli E Abbott G Mavilidi MF Galmes-Panades AM. Do physical fitness and executive function mediate the relationship between physical activity and academic achievement? An examination using structural equation modelling. Children. (2022) 9:823. doi: 10.3390/children9060823, 35740760 PMC9221993

[63] Ghorbanzadeh B Orangi BM Sahin T. The relationship between motor competence and executive function as influenced by age, sex and family socio-economic status. Front Psychol. (2025) 16:1544168. doi: 10.3389/fpsyg.2025.1544168, 40083765 PMC11903737

[64] Alonso-Cabrera J Salazar F Benavides-Ulloa J Parra-Rizo MA Zapata-Lamana R Diaz-Vargas C . Students from a public school in the south of Chile with better physical fitness markers have higher performance in executive functions tests: a cross-sectional study. Behav Sci. (2023) 13:191. doi: 10.3390/bs13020191. 36829420 PMC9951860

[65] Guo X Zhang Z Wei Q Lv H Wu R Zhao J. The relationship between obesity and neurocognitive function in Chinese patients with schizophrenia. BMC Psychiatry. (2013) 13:109. doi: 10.1186/1471-244X-13-109, 23570390 PMC3627610

[66] van der Niet AG Hartman E Smith J Visscher C. Modeling relationships between physical fitness, executive functioning and academic achievement in primary school children. Psychol Sport Exerc. (2014) 15:319–25. doi: 10.1016/j.psychsport.2014.02.010.

[67] Zhou Z Chen Y Huang K Zeng F Liang Z Wang N . Relationship between physical fitness and executive function in preschool children: a cross-sectional study. BMC Sports Sci Med Rehabil. (2024) 16:238. doi: 10.1186/s13102-024-01028-8, 39633414 PMC11616152

[68] Qin S Hermans EJ van Marle HJF Luo J Fernández G. Acute psychological stress reduces working memory-related activity in the dorsolateral prefrontal cortex. Biol Psychiatry. (2009) 66:25–32. doi: 10.1016/j.biopsych.2009.03.006, 19403118

[69] Takeuchi H Taki Y Hashizume H Sassa Y Nagase T Nouchi R . Failing to deactivate: the association between brain activity during a working memory task and creativity. NeuroImage. (2011) 55:681–7. doi: 10.1016/j.neuroimage.2010.11.052, 21111830

[70] Moreau D. Brains and brawn: complex motor activities to maximize cognitive enhancement. Educ Psychol Rev. (2015) 27:475–82. doi: 10.1007/s10648-015-9323-5

[71] Vestberg T Gustafson R Maurex L Ingvar M Petrovic P. Acute enhancement of executive functions through cognitively challenging physical activity games in elementary physical education. PLoS One. (2012) 7:e34731. doi: 10.1371/journal.pone.0034731, 22496850 PMC3319604

[72] Kolovelonis A Goudas M. Acute enhancement of executive functions through cognitively challenging physical activity games in elementary physical education. Eur Phys Educ Rev. (2023) 29:268–285. doi: 10.1177/1356336X221135139, 22496850

