# Supplementary material for: Comparative effects of physical education interventions on motor ability and executive function in junior high school students and their interrelationships
Source: Front Public Health. 2026 Apr 23;14:1779737. doi: 10.3389/fpubh.2026.1779737 (PMC13149061; doi:10.3389/fpubh.2026.1779737)
Supplement: Supplementary file 1 [file Data_Sheet_1.pdf]

Supplementary Material

1 Supplementary Tables

Table 1. Motor Cognition Scale - Item Summary

No	Item	Subdimension	Cronbach's α
1	I have mastered and can apply the learned motor skills.	Motor Cognition	0.967
2	I understand the cultural value and significance of the sport I have learned.		
3	I can apply the learned sport to daily exercise or competition.		
4	I am familiar with major domestic and international sporting events related to my chosen sport.		
5	I can briefly analyze phenomena and issues in sports competitions.		
6	I can apply my knowledge to watch and analyze sports competitions.		
7	I can revise my self-designed physical fitness plan under teacher guidance.		
8	I can accurately evaluate the effectiveness of my physical training.		

Table 2. Motor Skills Scale - Item Summary

Male Standards				Female Standards			
Performance (seconds)	Points	Performance (seconds)	Points	Performance (seconds)	Points	Time (seconds)	Points
7.5	75	9.78	37.5	8.5	75	10.79	37.5
7.62	73.5	9.83	36	8.62	73.5	10.84	36
7.74	72	9.88	34.5	8.74	72	10.89	34.5
7.86	70.5	9.93	33	8.86	70.5	10.94	33
7.98	69	9.98	31.5	8.98	69	10.99	31.5
8.1	67.5	10.03	30	9.1	67.5	11.04	30
8.22	66	10.08	28.5	9.22	66	11.09	28.5
8.34	64.5	10.13	27	9.34	64.5	11.14	27
8.46	63	10.18	25.5	9.46	63	11.19	25.5
8.58	61.5	10.23	24	9.58	61.5	11.24	24
8.7	60	10.28	22.5	9.7	60	11.29	22.5
8.82	58.5	10.33	21	9.82	58.5	11.34	21
8.94	57	10.38	19.5	9.94	57	11.39	19.5
9.18	55.5	10.43	18	10.06	55.5	11.44	18
9.23	54	10.48	16.5	10.24	54	11.49	16.5
9.28	52.5	10.53	15	10.29	52.5	11.54	15
9.33	51	10.58	13.5	10.34	51	11.59	13.5
9.38	49.5	10.63	12	10.39	49.5	11.64	12
9.43	48	10.68	10.5	10.44	48	11.69	10.5
9.48	46.5	10.73	9	10.49	46.5	11.74	9
9.53	45	10.78	7.5	10.54	45	11.79	7.5
9.58	43.5	10.83	6	10.59	43.5	11.84	6
9.63	42	10.88	4.5	10.64	42	11.89	4.5
9.68	40.5	10.93	3	10.69	40.5	11.94	3
9.73	39	10.98	1.5	10.74	39	11.99	1.5

Table 3. Main Effects Comparing the Impact of Different Instructional Methods and Gender on Motor Skill After Controlling for Pre-Test Consistency

Source	Type III sum of square	df	MS	F	p	η²
Corrected model	6.840[a]	6	1.14	2.796	0.019	0.234
Intercept	18.870	1	18.87	46.279	<.001	0.457
Pre-motor skill	3.545	1	3.545	8.695	0.005	0.137
Group	0.589	2	0.295	0.723	0.490	0.026
Gender	0.850	1	0.850	2.086	0.154	0.037
Group * Gender	1.023	2	0.512	1.255	0.293	0.044
Error	22.425	55	0.408			
Total	51.639	62				
Adj. Total	29.266	61				

df, Degrees of Freedom; MS, Mean Square.

Table 4. Main Effects Comparing the Impact of Different Instructional Methods and Gender on Motor Cognition After Controlling for Pre-Test Consistency

Source	Type III sum of square	df	MS	F	p	η²
Corrected model	11.777[a]	6	1.963	1.627	0.157	0.151
Intercept	9.131	1	9.131	7.566	0.008	0.121
Pre-motor cognition	1.257	1	1.257	1.042	0.312	0.019
Group	6.856	2	3.428	2.841	0.067	0.094
Gender	0.421	1	0.421	0.349	0.557	0.006
Group * Gender	0.334	2	0.167	0.138	0.871	0.005
Error	66.372	55	1.207			
Total	87.282	62				
Adj. Total	78.149	61				

df, Degrees of Freedom; MS, Mean Square.

Table 5. Main Effects Comparing the Impact of Different Instructional Methods and Gender on Stroop ACC After Controlling for Pre-Test Consistency

Source	Type III sum of square	df	MS	F	p	η²
Corrected model	.050[a]	6	0.008	0.412	0.868	0.043
Intercept	0.069	1	0.069	3.409	0.07	0.058
Pre-stroop ACC	0.001	1	0.001	0.039	0.845	0.001
Group	0.017	2	0.008	0.421	0.659	0.015
Gender	0.023	1	0.023	1.154	0.287	0.021
Group * Gender	0.006	2	0.003	0.139	0.871	0.005
Error	1.107	55	0.020			
Total	56.449	62				
Adj. Total	1.157	61				

df, Degrees of Freedom; MS, Mean Square.

Table 6. Main Effects Comparing the Impact of Different Instructional Methods and Gender on Stroop RT After Controlling for Pre-Test Consistency

Source	Type III sum of square	df	MS	F	p	η²
Corrected model	1260241.842[a]	6	210040.307	11.796	<.001	0.563
Intercept	276940.334	1	276940.334	15.553	<.001	0.220
Pre-stroop RT	1096184.517	1	1096184.517	61.563	<.001	0.528
Group	15258.085	2	7629.042	0.428	0.654	0.015
Gender	145.830	1	145.830	0.008	0.928	0.000
Group * Gender	46967.627	2	23483.813	1.319	0.276	0.046
Error	979327.895	55	17805.962			
Total	37644757.11	62				
Adj. Total	2239569.737	61				

df, Degrees of Freedom; MS, Mean Square.

Table 7. Main Effects Comparing the Impact of Different Instructional Methods and Gender on 2-back ACC After Controlling for Pre-Test Consistency.

Source	Type III sum of square	df	MS	F	p	η²
Corrected model	.048[a]	6	0.008	1.323	0.263	0.126
Intercept	0.128	1	0.128	21.084	<.001	0.277
Pre-2-back ACC	0.010	1	0.01	1.571	0.215	0.028
Group	0.017	2	0.009	1.413	0.252	0.049
Gender	0.012	1	0.012	1.907	0.173	0.034
Group * Gender	0.006	2	0.003	0.485	0.618	0.017
Error	0.334	55	0.006			
Total	48.624	62				
Adj. Total	0.382	61				

df, Degrees of Freedom; MS, Mean Square.

Table 8. Main Effects Comparing the Impact of Different Instructional Methods and Gender on 2-back RT After Controlling for Pre-Test Consistency.

Source	Type III sum of square	df	MS	F	p	η²
Corrected model	322809.609[a]	6	53801.602	2.268	0.05	0.198
Intercept	326260.632	1	326260.632	13.753	<.001	0.200
Pre-2-back RT	242507.785	1	242507.785	10.223	0.002	0.157
Group	4581.857	2	2290.929	0.097	0.908	0.003
Gender	488.398	1	488.398	0.021	0.886	0.000
Group * Gender	40724.128	2	20362.064	0.858	0.429	0.030
Error	1304724.454	55	23722.263			
Total	48883582.87	62				
Adj. Total	1627534.064	61				

df, Degrees of Freedom; MS, Mean Square.

Table 9. Main Effects Comparing the Impact of Different Instructional Methods and Gender on MOS ACC After Controlling for Pre-Test Consistency.

Source	Type III sum of square	df	MS	F	p	η²
Corrected model	.0560[a]	6	0.009	0.746	0.615	0.075
Intercept	0.469	1	0.469	37.581	<.001	0.406
Pre-MOS ACC	0.021	1	0.021	1.67	0.202	0.029
Group	0.012	2	0.006	0.477	0.623	0.017
Gender	0.012	1	0.012	0.953	0.333	0.017
Group * Gender	0.004	2	0.002	0.170	0.844	0.006
Error	0.687	55	0.012			
Total	50.648	62				
Adj. Total	0.743	61				

df, Degrees of Freedom; MS, Mean Square.
